# Oscillator decomposition of infant fNIRS data

**DOI:** 10.1371/journal.pcbi.1009985

**Published:** 2022-03-24

**Authors:** Takeru Matsuda, Fumitaka Homae, Hama Watanabe, Gentaro Taga, Fumiyasu Komaki

**Affiliations:** 1 RIKEN Center for Brain Science, RIKEN, Wako, Japan; 2 Department of Language Sciences, Tokyo Metropolitan University, Tokyo, Japan; 3 Research Center for Language, Brain and Genetics, Tokyo Metropolitan University, Tokyo, Japan; 4 Graduate School of Education, The University of Tokyo, Tokyo, Japan; 5 Graduate School of Information Science and Technology, The University of Tokyo, Tokyo, Japan; 6 International Research Center for Neurointelligence (IRCN), The University of Tokyo, Tokyo, Japan; Ghent University, BELGIUM

## Abstract

The functional near-infrared spectroscopy (fNIRS) can detect hemodynamic responses in the brain and the data consist of bivariate time series of oxygenated hemoglobin (oxy-Hb) and deoxygenated hemoglobin (deoxy-Hb) on each channel. In this study, we investigate oscillatory changes in infant fNIRS signals by using the oscillator decompisition method (OSC-DECOMP), which is a statistical method for extracting oscillators from time series data based on Gaussian linear state space models. OSC-DECOMP provides a natural decomposition of fNIRS data into oscillation components in a data-driven manner and does not require the arbitrary selection of band-pass filters. We analyzed 18-ch fNIRS data (3 minutes) acquired from 21 sleeping 3-month-old infants. Five to seven oscillators were extracted on most channels, and their frequency distribution had three peaks in the vicinity of 0.01-0.1 Hz, 1.6-2.4 Hz and 3.6-4.4 Hz. The first peak was considered to reflect hemodynamic changes in response to the brain activity, and the phase difference between oxy-Hb and deoxy-Hb for the associated oscillators was at approximately 230 degrees. The second peak was attributed to cardiac pulse waves and mirroring noise. Although these oscillators have close frequencies, OSC-DECOMP can separate them through estimating their different projection patterns on oxy-Hb and deoxy-Hb. The third peak was regarded as the harmonic of the second peak. By comparing the Akaike Information Criterion (AIC) of two state space models, we determined that the time series of oxy-Hb and deoxy-Hb on each channel originate from common oscillatory activity. We also utilized the result of OSC-DECOMP to investigate the frequency-specific functional connectivity. Whereas the brain oscillator exhibited functional connectivity, the pulse waves and mirroring noise oscillators showed spatially homogeneous and independent changes. OSC-DECOMP is a promising tool for data-driven extraction of oscillation components from biological time series data.

## Author summary

The functional near-infrared spectroscopy (fNIRS) can detect hemodynamic responses in the brain and the data consist of bivariate time series of oxygenated hemoglobin (oxy-Hb) and deoxygenated hemoglobin (deoxy-Hb) on each channel. In this study, we investigate oscillatory changes in fNIRS signals of infants by using a statistical method for extracting oscillators from time series data, which we call the oscillator decompisition method (OSC-DECOMP). OSC-DECOMP determines the number, frequencies and powers of oscillators as well as their projection patterns in a data-driven manner and does not require arbitrary selection of band-pass filters. Three types of oscillators (brain activity, pulse wave, mirroring noise) were found and each oscillator showed a characteristic spatial synchrony. Model comparison with the Akaike Information Criterion (AIC) demonstrated that the time series of oxy-Hb and deoxy-Hb on each channel originate from common oscillatory activity. We believe that OSC-DECOMP will become a promising tool of neural oscillation analysis for not only fNIRS but also LFP, EEG, MEG and fMRI.

## Introduction

Spontaneous changes in cerebral oxygenation reflect hemodynamic response to spontaneous neural activity. The functional Near-Infrared Spectroscopy (fNIRS) has been used to detect the low frequency oscillations of cerebral oxygenation with frequency range between 0.01 and 0.1 Hz in adults [1–4], elders [5] and infants [6]. Temporal correlation of the oscillatory signals measured at multiple locations revealed the functional connectivity of the cortex in adults [7–9] and infants [10]. However, there are three outstanding problems. (1) In addition to neurogenic changes, hemodynamic oscillations include cardiovascular oscillations such as cardiac (-1 Hz), respiratory (-0.3 Hz), Mayer wave (-0.1 Hz), and vasomotion (-0.1 Hz) [1, 3, 4, 11, 12]. Conventionally, such oscillation components in fNIRS are extracted by applying band-pass filters to the raw data. (2) Although signals with a wide frequency band (0.01–0.1 Hz) have been used to analyze functional connectivity, subdivision of the frequency range revealed that distinct functional connectivity is dependent on a specific frequency range [13]. It is not clear which frequency range is involved in the functional connectivity in different behavioral states such as wake and sleep, and different populations such as infants and the elderly. (3) The fNIRS can detect both oxygenated and deoxygenated hemoglobin (oxy- and deoxy-Hb) with high temporal resolution (-10 ms). The phase relationship between oxy- and deoxy-Hb changes is referred to as hemoglobin phase of oxygenation and deoxygenation (hPod) and provides rich information about cerebral hemodynamics and metabolism [14]. The phase of the time series has been computed by the Hilbert transform to bandpass-filtered data [6, 14, 15]. However, the selection of the appropriate frequency of the bandpass filter is not clear. It is also not clear whether multivariate time series such as oxy- and deoxy-Hb signals originate from a common oscillator or independent oscillators.

**Fig 1:**
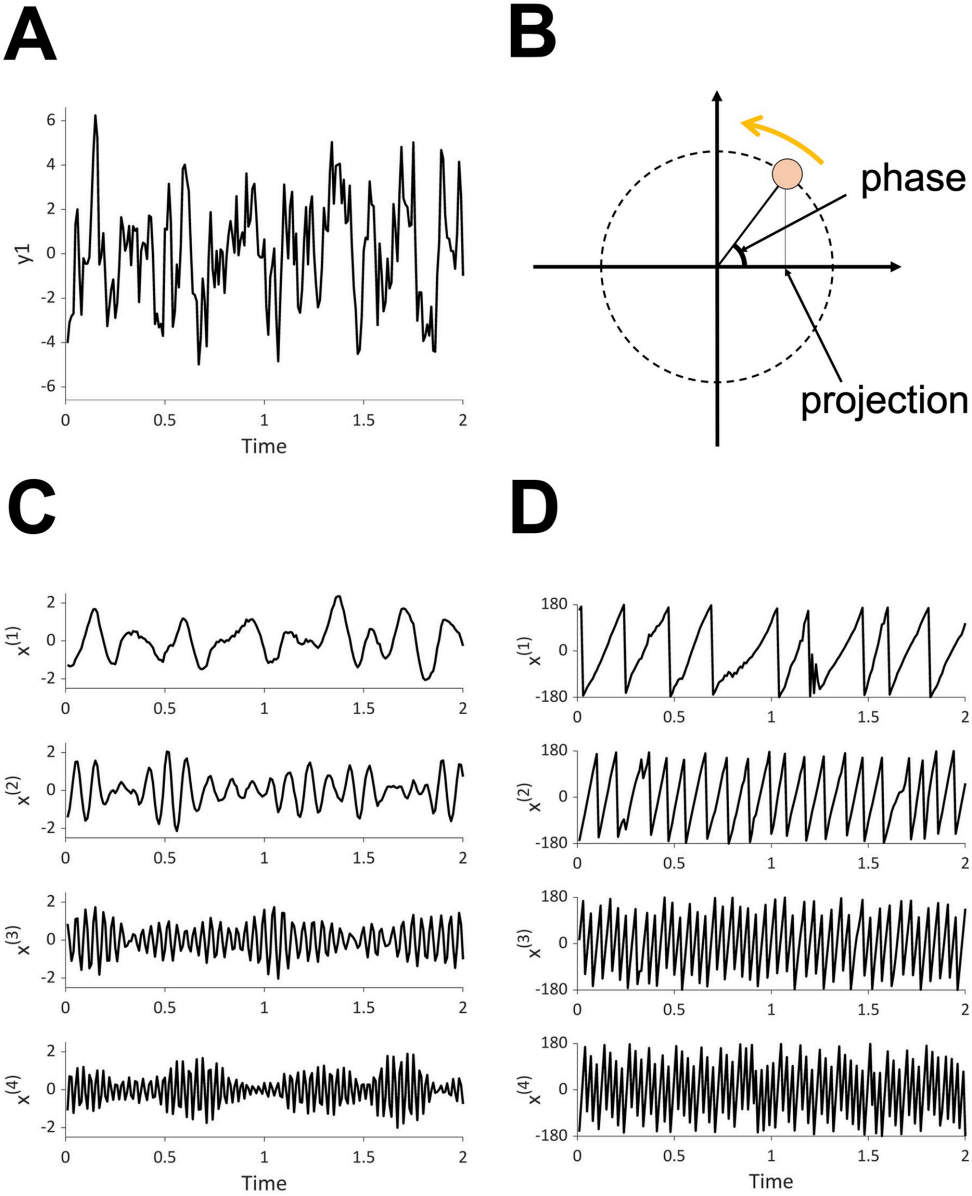
OSC-DECOMP for univariate time series data. (A) Input data. (B) Schematic of OSC-DECOMP. Each oscillation component is the projection onto the horizontal axis (first coordinate) of an oscillator that rotates on a plane with random fluctuation. (C) Decomposition into four oscillation components. (D) Phase of four oscillators (in degrees).

Recently, a statistical method for extracting oscillators from time series data was developed [16, 17], called the oscillator decompisition method (OSC-DECOMP). Here, we briefly demonstrate OSC-DECOMP using figures. Its mathematical detail will be explained in Section 2.3. Fig 1 shows an example of application to univariate time series data. In this case, OSC-DECOMP determines that the given time series (Fig 1A) is a superposition of four oscillation components (Fig 1C) plus noise, based on statistical model fitting. More precisely, each oscillation component in Fig 1C is the projection onto the horizontal axis (first coordinate) of an oscillator that rotates on a plane with random fluctuation (Fig 1B), and OSC-DECOMP extracts an appropriate number of oscillators from the time series. Thus, OSC-DECOMP also provides the phase of each oscillator (Fig 1D). In this way, OSC-DECOMP enables data-driven investigation of the oscillatory dynamics that underlie the time series data. Notably, OSC-DECOMP determines the number and the frequencies of the underlying oscillators in a data-driven (objective) manner and does not require the arbitrary selection of band-pass filters.

**Fig 2:**
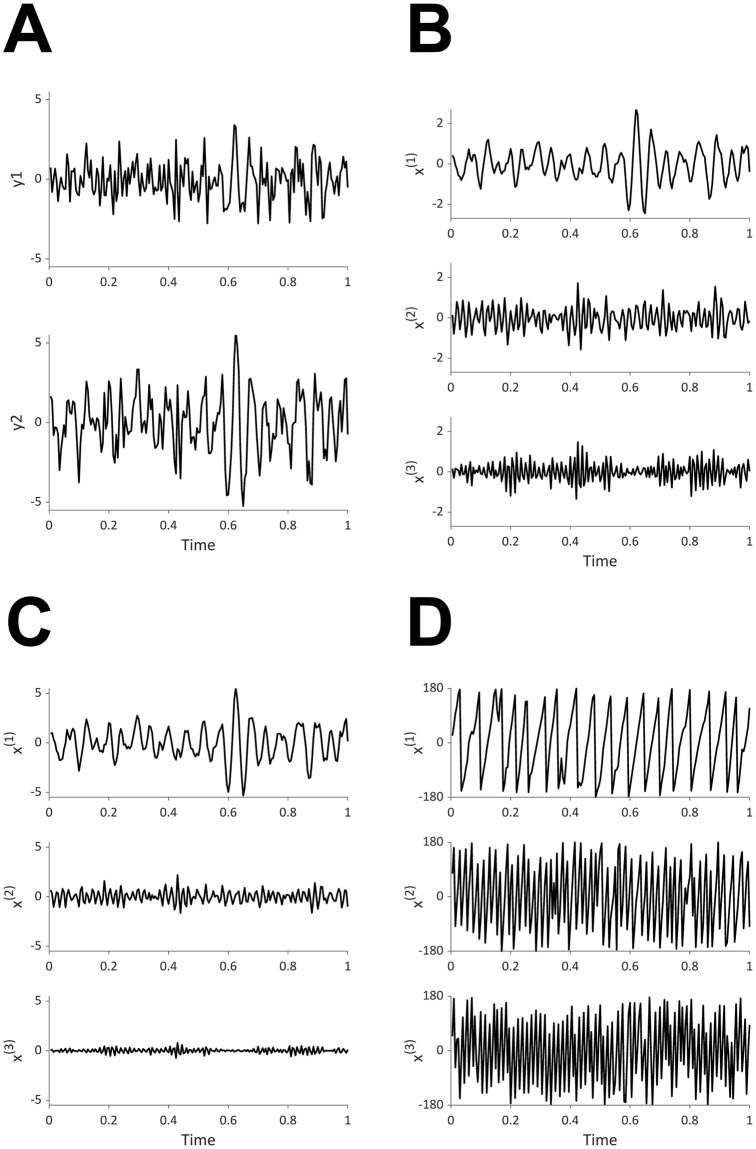
OSC-DECOMP for bivariate time series data. (A) Input data. (B) Decomposition into three oscillation components of the first time series. (C) Decomposition into three oscillation components of the second time series. Although the waveform may look almost the same, the scale is different from (B). (D) Phase of extracted oscilators (in degrees).

**Fig 3:**
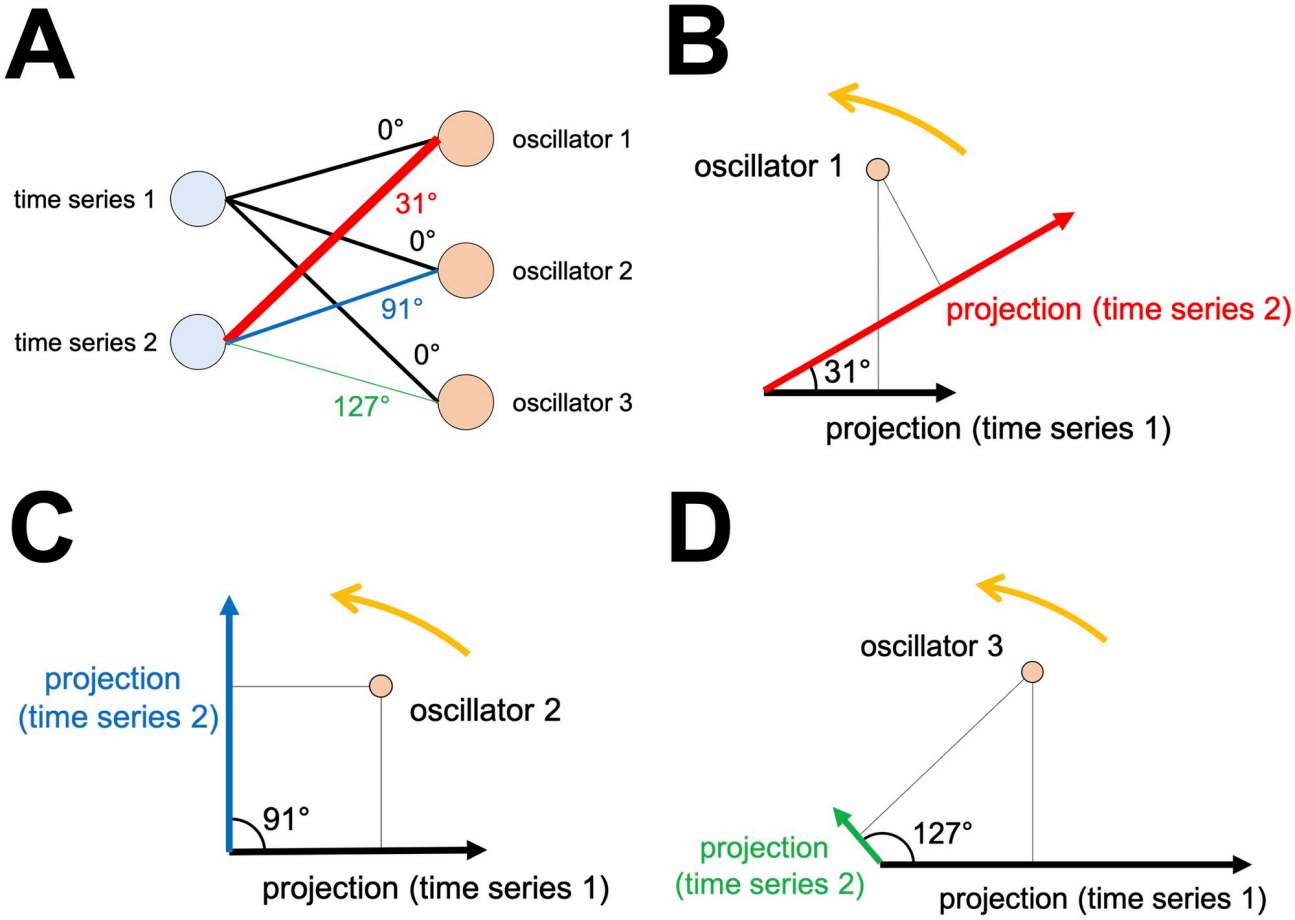
Estimated projection pattern between each time series and each oscillator. (A) Bipartite graph representation. The thickness of each line indicates the amplitude modulation. The color and text of each line represent the phase modulation. (B) (C) (D) The projection pattern for three oscillators.

OSC-DECOMP is also applicable to multivariate time series data [17]. Fig 2 shows an example of application to bivariate time series data. In this case, OSC-DECOMP establishes that the given bivariate time series (Fig 2A) originates from three oscillators. As a result, each time series is decomposed into three oscillation components (Fig 2B and 2C), and the phase of the extracted oscillators is also obtained (Fig 2D). The oscillation components in Fig 2B and 2C are the projection of the extracted oscillators. However, unlike the case of the univariate time series (Fig 1B), when applied to multivariate time series, OSC-DECOMP estimates the projection pattern between each time series and each oscillator based on data, and the projection pattern describes the amplitude and phase modulation. Fig 3 shows the estimated projection pattern. In Fig 3A, the thickness of each line represents the amplitude modulation, whereas the color and text of each line indicate the phase modulation. For example, Fig 3B shows the projection pattern for the first oscillator, in which the black and red vectors correspond to the first and second time series, respectively. Given that the red vector is longer than the black vector, the first oscillator superposes on the second time series more than on the first time series. On the other hand, since the angle between the two vectors is 31°, the second time series has a 31° phase delay compared to the first time series. Similarly, Fig 3C and 3D presents the projection pattern for the second and third oscillators. In this way, the projection pattern is estimated in the form of vectors. Thus, OSC-DECOMP enables the investigation of the common oscillators underlying multivariate time series in a data-driven manner.

In this study, we investigated oscillatory changes in infant fNIRS data by using OSC-DECOMP. Specifically, we analyzed fNIRS data (3 minutes) acquired from sleeping 3-month-old infants [10] and extracted oscillators on each channel of each infant. The phase difference between oxy- and deoxy-Hb was then calculated for each oscillator. By comparing the Akaike Information Criterion (AIC) of two state space models, we determined that the time series of oxy- and deoxy-Hb on each channel originate from common oscillatory activity. We also utilized the result of OSC-DECOMP to investigate frequency-specific functional connectivity.

## Material and methods

### Ethics statement

In the present study, we analyze data that was previously reported in [10, 18]. The Office for Life Science Research Ethics and Safety, the University of Tokyo, approved this study (No. 20–225). The parent(s) of all the infants provided written informed consent prior to initiation of experiments.

### Participants

We analyzed the data obtained from 21 full-term infants (11 girls and 10 boys; mean postnatal age: 111.6 days, range: 102–123 days) as they slept naturally.

### Data acquisition

**Fig 4:**
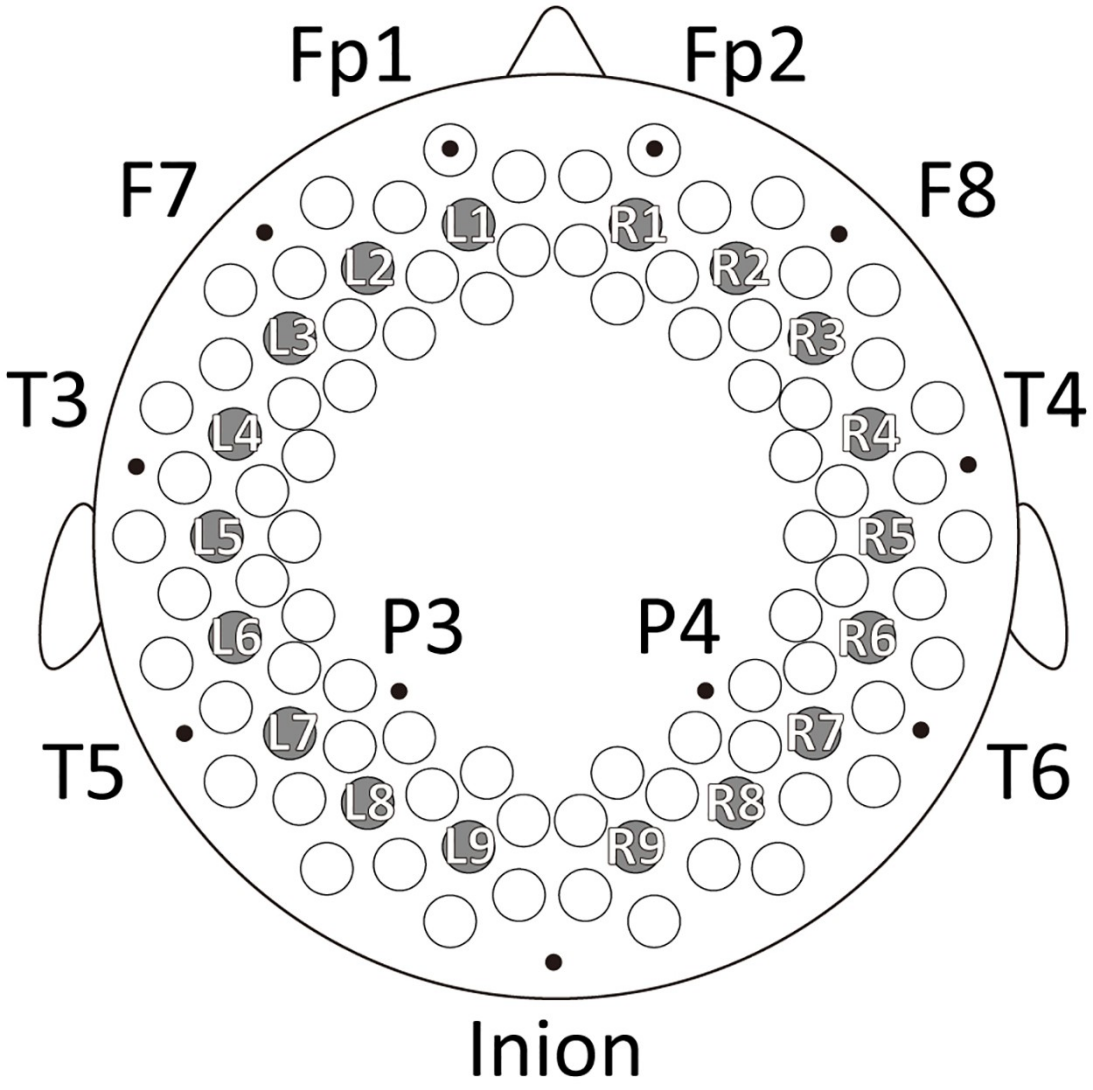
Arrangement of fNIRS measurement channels. Circles indicate 94 measurement channels. Eighteen circles filled in gray (L1 to L9 and R1 to R9) show the channels used in the present analysis. For reference, some landmarks (black dots) of the international 10/20 system are shown.

We used an fNIRS instrument (ETG-7000; Hitachi Medical Corporation) with 94 measurement channels (47 channels in each hemisphere) to detect the relative concentration changes in oxy-Hb and deoxy-Hb [millimolar-millimeter (mM ⋅ mm)] for 3 min without presenting external stimuli. The sampling rate was set at 10 Hz, resulting in 1,800 points per channel of spontaneous changes in oxy- and deoxy-Hb signals. Two sets of 3 × 10 array probes composed of 15 sources and 15 detectors of NIR light (wavelengths: 785 nm and 830 nm, intensity: 0.6 mW), which defined 47 channels, were mounted on a flexible cap over the frontal, temporal, parietal, and occipital regions of the left and right hemispheres. The distance between the source and detector was set to approximately 2.0 cm. The international 10/20 system of electrode placement was referenced to set the positions of the measurement channels. We previously estimated the locations of channels using a magnetic resonance imaging brain atlas [15, 19]. To evaluate the tendency in broad cortical regions and reduce computational complexity, we selected data from nine channels located in the middle line of the array on each hemisphere for the following analyses (Fig 4).

### Oscillator decompisition method (OSC-DECOMP)

To extract the underlying oscillators from the bivariate time series of oxy- and deoxy-Hb on each channel, we applied the oscillator decompisition method (OSC-DECOMP) [16, 17]. The MATLAB code is available online at http://www.stat.t.u-tokyo.ac.jp/~t-matsuda/software.html. Here, we briefly review this method. See S1 Appendix for background on state space models and time series decomposition.

Let *y* = (*y*_*t*,*j*_) (*t* = 1, …, *N*; *j* = 1, …, *J*) be a *J*-dimensional time series data of length *N* with sampling period Δ*t*. The current fNIRS data corresponds to *J* = 2, *N* = 1800 and Δ*t* = 0.1 s. OSC-DECOMP assumes that there are *K* oscillators underlying *y*, each of which is represented by a point on a two-dimensional plane that rotates around the origin with random fluctuation (Fig 1B). Let $\left(x_{t,1}^{\left(k\right)},x_{t,2}^{\left(k\right)}\right)$ be the coordinate of the *k*-th oscillator at time *t*. Then, the stochastic dynamics of this oscillator is modeled as

$$\begin{array}{c} (\begin{array}{c} x_{t+1,1}^{\left(k\right)}\\ x_{t+1,2}^{\left(k\right)} \end{array})=a_{k}(\begin{array}{cc} \text{cos}(2\pi f_{k}\Delta t) & -\text{sin}(2\pi f_{k}\Delta t)\\ \text{sin}(2\pi f_{k}\Delta t) & \text{cos}(2\pi f_{k}\Delta t) \end{array})(\begin{array}{c} x_{t,1}^{\left(k\right)}\\ x_{t,2}^{\left(k\right)} \end{array})+(\begin{array}{c} v_{t,1}^{\left(k\right)}\\ v_{t,2}^{\left(k\right)} \end{array}), \end{array}$$ (1)

where 0 < *a*_*k*_ < 1, 0 ≤ 2*πf*_*k*_Δ*t* ≤ *π* and $\left(v_{t,1}^{\left(k\right)},v_{t,2}^{\left(k\right)}\right)$ is an isotropic Gaussian noise with mean zero and variance $\sigma_{k}^{2}$:

$$\begin{array}{c} (\begin{array}{c} v_{t,1}^{\left(k\right)}\\ v_{t,2}^{\left(k\right)} \end{array})∼N((\begin{array}{c} 0\\ 0 \end{array}),(\begin{array}{cc} \sigma_{k}^{2} & 0\\ 0 & \sigma_{k}^{2} \end{array})). \end{array}$$

Since the 2 × 2 matrix in the right hand side of (1) represents the rotation through an angle 2*πf*_*k*_Δ*t* about the origin, the parameter *f*_*k*_ is interpreted as the frequency of the *k*-th oscillator. The parameter *a*_*k*_ is viewed as the regularity of the *k*-th oscillator in the sense that the waveform of $x_{t,1}^{\left(k\right)}$ (or $x_{t,2}^{\left(k\right)}$) is closer to the sinusoidal curve for *a*_*k*_ closer to one. Also, the parameter $\sigma_{k}^{2}$ specifies the power of the *k*-th oscillator because the stationary variance of $x_{t,1}^{\left(k\right)}$ (or $x_{t,2}^{\left(k\right)}$) is $\sigma_{k}^{2}/\left(1-a_{k}^{2}\right)$. The phase of the *k*-th oscillator is defined by $\text{arg}(x_{t,1}^{\left(k\right)}+x_{t,2}^{\left(k\right)}\sqrt{-1})$, where arg(*z*) is the argument of the complex number *z* (Fig 1B).

By using the *K* oscillators, the observed time series *y* = (*y*_*t*,*j*_) is modeled as

$$\begin{array}{c} y_{t,j}=\sum_{k=1}^{K}\left(c_{jk,1}x_{t,1}^{\left(k\right)}+c_{jk,2}x_{t,2}^{\left(k\right)}\right)+w_{t,j}, \end{array}$$ (2)

where $w_{t,j}∼N\left(0,\tau^{2}\right)$ are independent Gaussian noise. Namely, the observation *y*_*t*,*j*_ is assumed to be the sum of the *K* inner products of the vectors (*c*_*jk*,1_, *c*_*jk*,2_) with the oscillator coordinates $\left(x_{t,1}^{\left(k\right)},x_{t,2}^{\left(k\right)}\right)$ and observation noise *w*_*t*,*j*_. Recall that the inner product *A* ⋅ *B* of two vectors *A* and *B* is given by

$$\begin{array}{c} A\cdot B=\|A\|\cdot \|B\|\cdot \text{cos}\;\phi , \end{array}$$

where ‖*A*‖ is the length (norm) of *A* and *ϕ* is the angle between *A* and *B*. Therefore, the observation model (2) means that the *k*-th oscillator is superposed on the *j*-th time series with amplitude multiplied by ${\left(c_{jk,1}^{2}+c_{jk,2}^{2}\right)}^{1/2}$ and phase delayed by arg(*c*_*jk*,1_ + *ic*_*jk*,2_) (Fig 2E and 2F). In order to ensure parameter identifiability, we fix (*c*_1*k*,1_, *c*_1*k*,2_) = (1, 0) for *k* = 1, …, *K*.

The pair (1) and (2) forms a Gaussian linear state space model [20, 21]. Therefore, the posterior distributions of the oscillator coordinates $\left(x_{t,1}^{\left(k\right)},x_{t,2}^{\left(k\right)}\right)$ given *y*_1_, …, *y*_*N*_ are Gaussian and their mean and covariance can be computed by the Kalman smoother algorithm (see S1 Appendix for details). Thus, we apply the Kalman smoother to compute the time series of *K* oscillators with credible intervals. To extract oscillators in a data-driven manner, the model parameter

$$\begin{array}{c} \theta_{K}=\left(a_{1},\ldots ,a_{K},f_{1},\ldots ,f_{K},\sigma_{1}^{2},\ldots ,\sigma_{K}^{2},c_{21,1},c_{21,2},\ldots ,c_{JK,1},c_{JK,2},\tau^{2}\right) \end{array}$$

is estimated by the maximum marginal likelihood:

$$\begin{array}{c} {\hat{\theta }}_{K}=\text{arg}\;\underset{\theta_{K}}{\text{max}}\;\text{log}\;p\left(y_{1}\ldots ,y_{N}|\theta_{K}\right). \end{array}$$

The confidence intervals can be also constructed. See S1 Appendix for details. In addition, the number of oscillators *K* is determined by minimizing the Akaike Information Criterion (AIC) [22]. Specifically, we fit the state space model (1) and (2) with *K* = 1, …, *K*_max_ and then select *K* by

$$\begin{array}{c} \hat{K}=\text{arg}\;\underset{K}{\text{min}}\;\text{AIC}\left(K\right), \end{array}$$

where

$$\begin{array}{c} \text{AIC}\left(K\right)=-2\;\text{log}\;p\left(y_{1}\ldots ,y_{N}|{\hat{\theta }}_{K}\right)+2\left(\left(J+2\right)K+1\right). \end{array}$$

### Testing of common oscillator hypothesis

We fitted the state space model (1) and (2) with *J* = 2 to the bivariate time series of oxy- and deoxy-Hb on each channel. This model is based on the assumption that the two time series originate from common oscillators. Whereas this assumption seems reasonable for the fNIRS data, there is another possibility that each time series originates from its own oscillators and thus develops independently with each other. Namely, we can consider the state space model (1) and (2) with *J* = 1 [16] for each time series, which provides another model for the fNIRS data. Thus, we tested the null hypothesis that these two time series models have the same goodness-of-fit to the data. We used the following Linhart-type test [23] to determine the significance of the AIC difference.

A statistical model for time series data *y*_1_, …, *y*_*N*_ is given by a probability distribution

$$\begin{array}{c} p\left(y_{1},\ldots ,y_{N}|\theta \right)=p\left(y_{1}|\theta \right)p\left(y_{2}|y_{1},\theta \right)\cdots p\left(y_{N}|y_{1},\ldots ,y_{N-1},\theta \right), \end{array}$$

where *θ* is an unknown parameter to be estimated from data. Consider two candidate models *p*^(1)^(*y*_1_, …, *y*_*N*_ ∣ *θ*^(1)^) and *p*^(2)^(*y*_1_, …, *y*_*N*_ ∣ *θ*^(2)^). Let ${\hat{\theta }}^{\left(1\right)}$ and ${\hat{\theta }}^{\left(2\right)}$ be the maximum likelihood estimates of *θ*^(1)^ and *θ*^(2)^, respectively. Then, the AIC of these models are

$$\begin{array}{c} \text{AIC}\left(1\right)=-2\;\text{log}\;p^{\left(1\right)}\left(y_{1},\ldots ,y_{N}|\hat{\theta^{\left(1\right)}}\right)+2\cdot \text{dim}\left(\theta^{\left(1\right)}\right),\\ \text{AIC}\left(2\right)=-2\;\text{log}\;p^{\left(2\right)}\left(y_{1},\ldots ,y_{N}|\hat{\theta^{\left(2\right)}}\right)+2\cdot \text{dim}\left(\theta^{\left(2\right)}\right). \end{array}$$

Let

$$\begin{array}{c} l_{t}^{\left(1\right)}=\text{log}\;p^{\left(1\right)}\left(y_{t}|y_{1},\ldots ,y_{t-1},{\hat{\theta }}^{\left(1\right)}\right),\\ l_{t}^{\left(2\right)}=\text{log}\;p^{\left(2\right)}\left(y_{t}|y_{1},\ldots ,y_{t-1},{\hat{\theta }}^{\left(2\right)}\right), \end{array}$$

be the log-likelihood of one-step ahead prediction, where $l_{1}^{\left(1\right)}=p^{\left(1\right)}\left(y_{1}|{\hat{\theta }}^{\left(1\right)}\right)$ and $l_{1}^{\left(2\right)}=p^{\left(2\right)}\left(y_{1}|{\hat{\theta }}^{\left(2\right)}\right)$ are defined from the stationary distributions of the models. Motivated by Linhart’s test [23], we construct the test statistic as

$$\begin{array}{c} z=\frac{1}{2\sqrt{N}}\frac{\text{AIC}(1)-\text{AIC}(2)}{{\left(\Sigma_{11}+\Sigma_{22}-2\Sigma_{12}\right)}^{1/2}}, \end{array}$$

where

$$\begin{array}{c} \mu =\frac{1}{N}\sum_{t=1}^{N}(\begin{array}{c} l_{t}^{\left(1\right)}\\ l_{t}^{\left(2\right)} \end{array}),\;\Sigma =\frac{1}{N}\sum_{t=1}^{N}(\begin{array}{cc} {\left(l_{t}^{\left(1\right)}-\mu_{1}\right)}^{2} & \left(l_{t}^{\left(1\right)}-\mu_{1}\right)\left(l_{t}^{\left(2\right)}-\mu_{2}\right)\\ \left(l_{t}^{\left(1\right)}-\mu_{1}\right)\left(l_{t}^{\left(2\right)}-\mu_{2}\right) & {\left(l_{t}^{\left(2\right)}-\mu_{2}\right)}^{2} \end{array}). \end{array}$$

Under the null hypothesis that the two models have the same goodness-of-fit to the data, the distribution of *z* is approximately the standard normal N(0, 1). Therefore, the p-value is calculated as

$$\begin{array}{c} p=2(1-\Phi (|z|)), \end{array}$$

where Φ is the cumulative distribution function of the standard normal distribution. Thus, the null hypothesis is rejected if *p* is smaller than the significance level *α*. For verification, we also applied the Wilcoxon signed-rank test to $\left(l_{1}^{\left(1\right)},\ldots ,l_{N}^{\left(1\right)}\right)$ and $\left(l_{1}^{\left(2\right)},\ldots ,l_{N}^{\left(2\right)}\right)$, which does not require the normality assumption on *z*.

### Frequency-specific functional connectivity

Finally, we investigated the frequency-specific functional connectivity based on the result of OSC-DECOMP.

As a result of OSC-DECOMP, we obtained a bivariate time series for each oscillator. To evaluate the correlation between two oscillators, we employed canonical correlation analysis, which is a statistical method for investigating the relationship between two sets of random variables. The (first) canonical correlation coefficient between two random vectors $X\in R^{n}$ and $Y\in R^{m}$ is defined as

$$\begin{array}{c} r=\underset{\begin{array}{c} a\in R^{n}\\ b\in R^{m} \end{array}}{\text{max}}\;\text{Corr}\left[a^{⊤}X,b^{⊤}Y\right], \end{array}$$

where Corr denotes the Pearson correlation coefficient and (*a*, *b*) ≠ (0, 0). This problem is reduced to the generalized eigenvalue problem:

$$\begin{array}{c} (\begin{array}{cc} O & \Sigma_{XY}\\ \Sigma_{YX} & O \end{array})(\begin{array}{c} a\\ b \end{array})=2r(\begin{array}{cc} \Sigma_{XX} & O\\ O & \Sigma_{YY} \end{array})(\begin{array}{c} a\\ b \end{array}), \end{array}$$

where Σ_*XX*_, Σ_*XY*_, Σ_*YX*_, Σ_*YY*_ are submatrices of the covariance matrix of (*X*, *Y*), given by

$$\begin{array}{c} \text{Cov}\left[\left(X,Y\right),\left(X,Y\right)\right]=(\begin{array}{cc} \Sigma_{XX} & \Sigma_{XY}\\ \Sigma_{YX} & \Sigma_{YY} \end{array}). \end{array}$$

The canonical correlation coefficient *r* is regarded as a measure of the linear dependence between *X* and *Y*.

To determine the functional connectivity for target frequency bands, we first selected an oscillator with a frequency *f*_*k*_ in the target frequency band on each channel. If there was more than one oscillator in the target frequency band, we selected the oscillator with the maximum power. Next, for each pair of channels, we calculated the (first) canonical correlation coefficient between the selected oscillators. Namely, we substituted the empirical covariance matrix of the oscillators into the preceding formula. We then considered the channel pairs with a canonical correlation coefficient that exceeded the threshold as functionally connected. Thus, we obtained a network of frequency-specific functional connectivity.

## Results

### Oscillator decomposition of synthetic data

Before presenting the results on real data, we first validate the performance of OSC-DECOMP on synthetic data with comparison to the conventional method with bandpass filtering. For more experiments, see [16, 17].

We generated bivariate time series from the state space model (1) and (2) with *J* = 2, *K* = 3, *N* = 1800, Δ*t* = 0.1s and

$$\begin{array}{cc} \left(a_{1},a_{2},a_{3}\right) & =\left(0.99,0.99,0.4\right),\;\left(f_{1},f_{2},f_{3}\right)=\left(0.03,2,2\right),\\ \left(\sigma_{1}^{2},\sigma_{2}^{2},\sigma_{3}^{2}\right) & =(0.01\left(1-a_{1}^{2}\right),0.01\left(1-a_{2}^{2}\right),0.01\left(1-a_{3}^{2}\right)),\\ \left(c_{21,1},c_{21,2}\right) & =(0.5\;\text{cos}\;230^{\circ },0.5\;\text{sin}\;230^{\circ }),\\ \left(c_{22,1},c_{22,2}\right) & =\left(-1,0\right),\;\left(c_{23,1},c_{23,2}\right)=\left(0.2,0\right),\;\tau^{2}=10^{-6}. \end{array}$$

**Fig 5:**
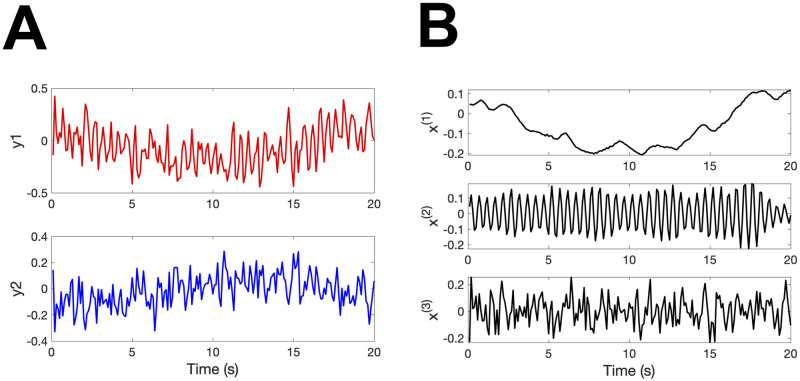
(A) Synthetic data. (B) Estimated oscillation components.

This parameter setting is motivated from the property of infant fNIRS data found in the next subsection. For this data, OSC-DECOMP correctly detected $\hat{K}=3$ oscillators by minimizing AIC and their frequencies were ${\hat{f}}_{1}=0.0343$ Hz, ${\hat{f}}_{2}=2.0016$ Hz and ${\hat{f}}_{3}=2.0421$ Hz. Fig 5 summarizes the result.

Conventionally, oscillation components in time series data are extracted by using the bandpass filters. Here, we compare the accuracy of OSC-DECOMP and bandpass filtering in reconstructing the first oscillation component (time series of the first coordinate of the first oscillator). Note that the second and third oscillators are difficult to separate by the bandpass filter because their spectra significantly overlap. For bandpass filtering, we used the MATLAB function *bandpass* with passband 0.01–0.1 Hz. The mean squared error in reconstructing the first oscillation component was 5.1 × 10^−4^ for OSC-DECOMP and 5.6 × 10^−3^ for bandpass filtering. Thus, OSC-DECOMP extracts the oscillation component more accurately than bandpass filtering.

We also compare the accuracy of OSC-DECOMP and bandpass filtering in estimating the phase difference between two time series (e.g. oxy- and deoxy-Hb). For the same reason as the previous paragraph, we focus on the phase difference for the first oscillator, which is 230°. For OSC-DECOMP, the estimate of the projection vector was $\left({\hat{c}}_{21,1},{\hat{c}}_{21,2}\right)=\left(-0.275,-0.417\right)$ and thus the estimate of the phase difference is $\text{arg}\left({\hat{c}}_{21,1}+\sqrt{-1}{\hat{c}}_{21,2}\right)=236.54^{\circ }$. In addition, by using the Hessian of the negative log-likelihood at the maximum likelihood estimate (observed Fisher information [24]) and the delta method [25], the 95% confidence interval of the phase difference was obtained as [230.03°, 243.05°]. We used an extension of the Kalman filter algorithm to compute the Hessian [26]. See S1 Appendix for details. For bandpass filtering, the phase difference in 0.01–0.1 Hz at every time point was computed by applying the Hilbert transform to the filtered signals. From these samples of angular variables, the point estimate and 95% confidence interval of the mean direction were computed by using the MATLAB toolbox CircStat [27], which implements the usual procedures for the von Mises distribution [28]. The results were 209.86° and [205.70°, 214.02°]. Thus, OSC-DECOMP provides more accurate point estimates and confidence intervals of the phase difference than bandpass filtering.

**Fig 6:**
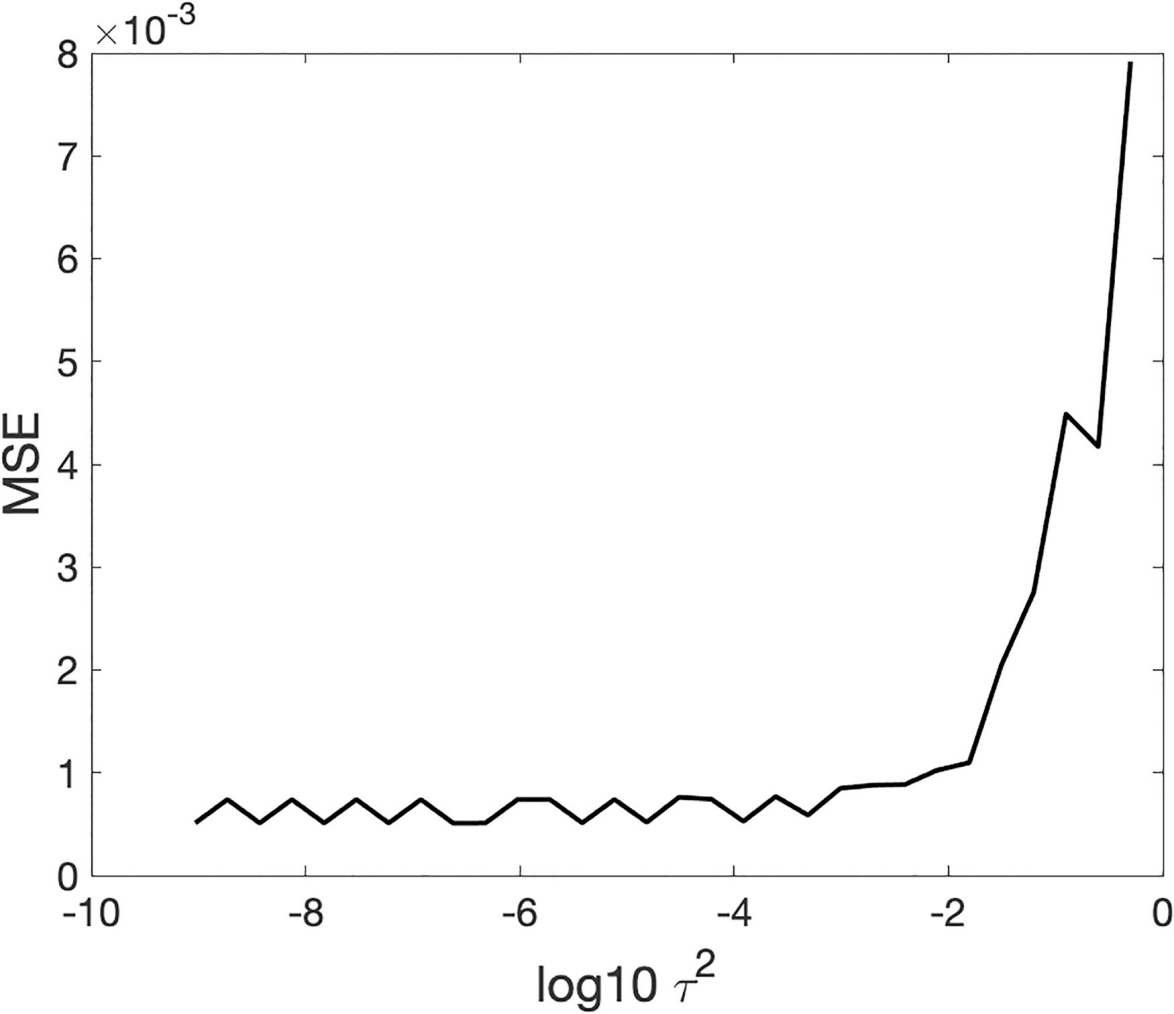
Noise sensitivity of OSC-DECOMP.

Fig 6 shows the noise sensitivity of OSC-DECOMP. It plots the mean squared error of OSC-DECOMP in estimating the first oscillation component with respect to the variance *τ*^2^ of observation noise. It demonstrates that the estimation accuracy of OSC-DECOMP is almost constant as long as *τ*^2^ is smaller than 10^−3^.

### Oscillator decomposition of infant fNIRS data

To investigate the oscillatory changes in the 18-ch fNIRS data (3 minutes) taken from 21 sleeping 3-month-old infants, we applied OSC-DECOMP to the bivariate time series of oxy- and deoxy-Hb on each channel.

**Fig 7:**
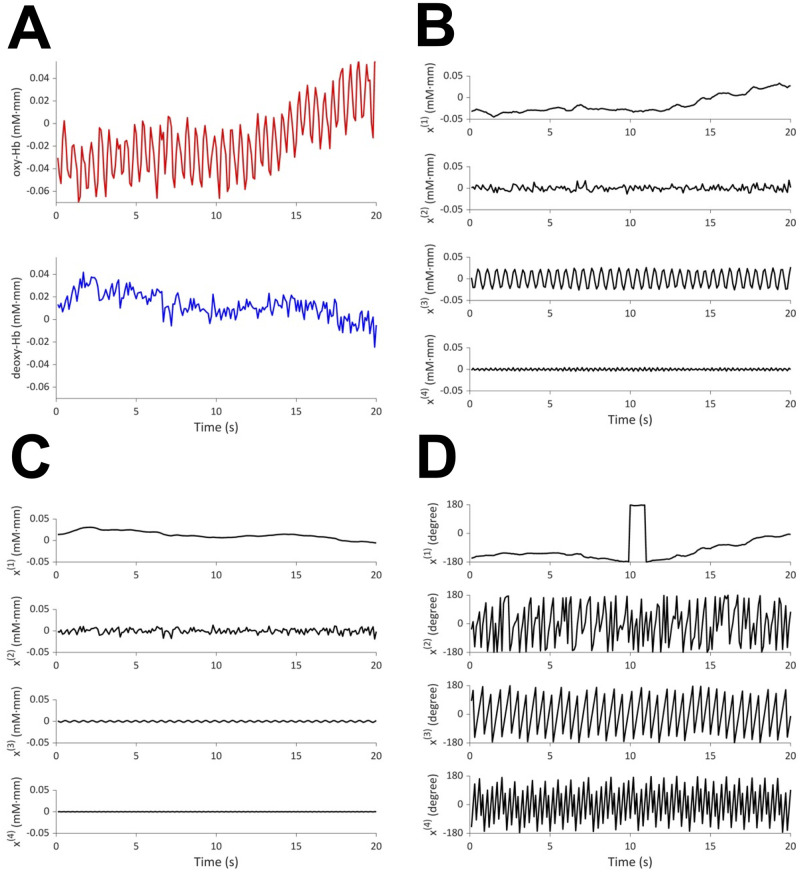
(A) Infant fNIRS (20 seconds). Red: oxy-Hb; blue: deoxy-Hb. (B) Four oscillation components in oxy-Hb. (C) Four oscillation components in deoxy-Hb. (D) Phase of the extracted oscillators.

**Fig 8:**
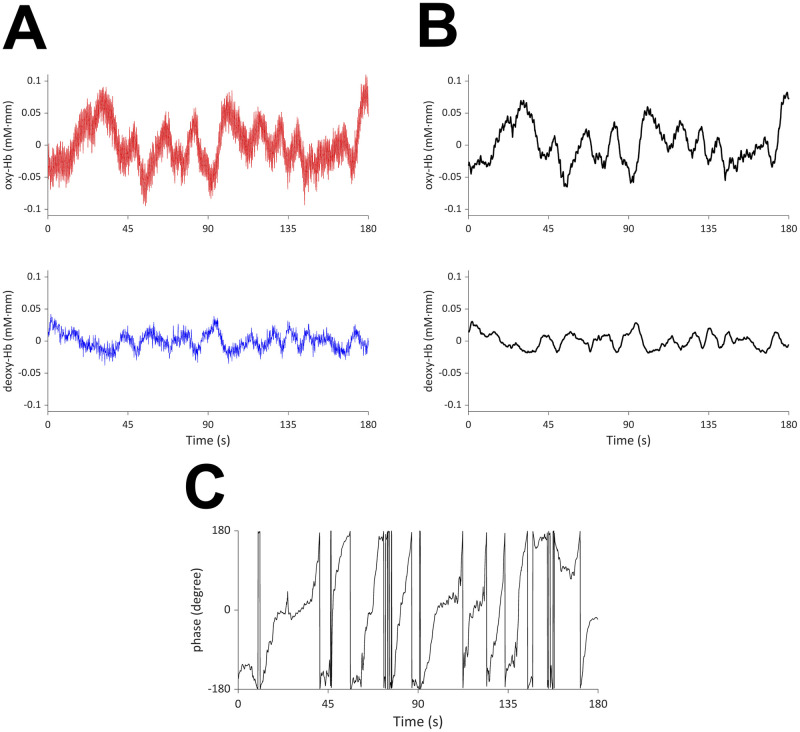
(A) Infant fNIRS (3 minutes). Red: oxy-Hb; blue: deoxy-Hb. (B) First oscillation component in oxy- (upper) and deoxy-Hb (lower). (C) Phase of the first oscillator.

**Fig 9:**
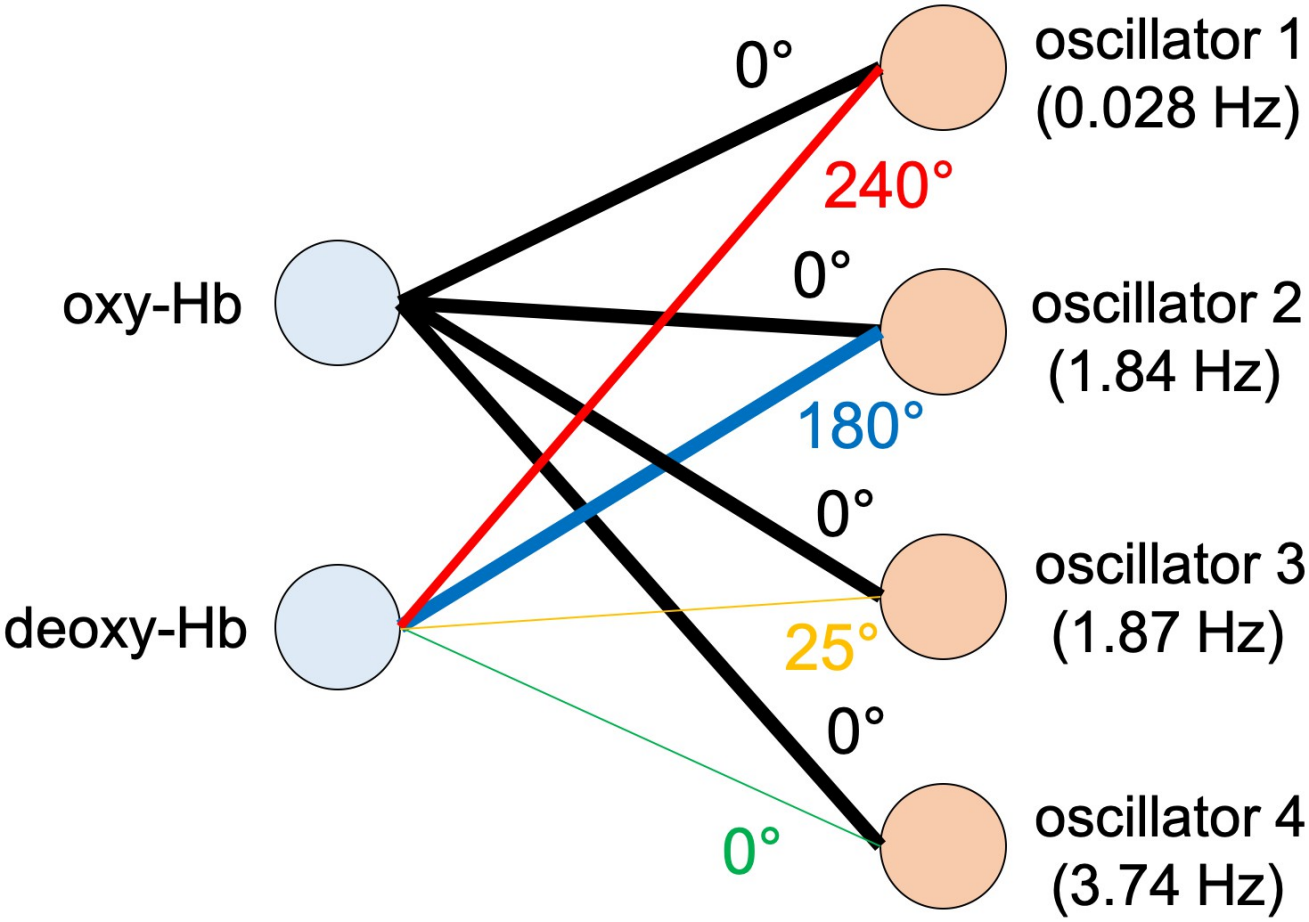
Estimated projection pattern for infant fNIRS data in Figs 7 and 8.

**Fig 10:**
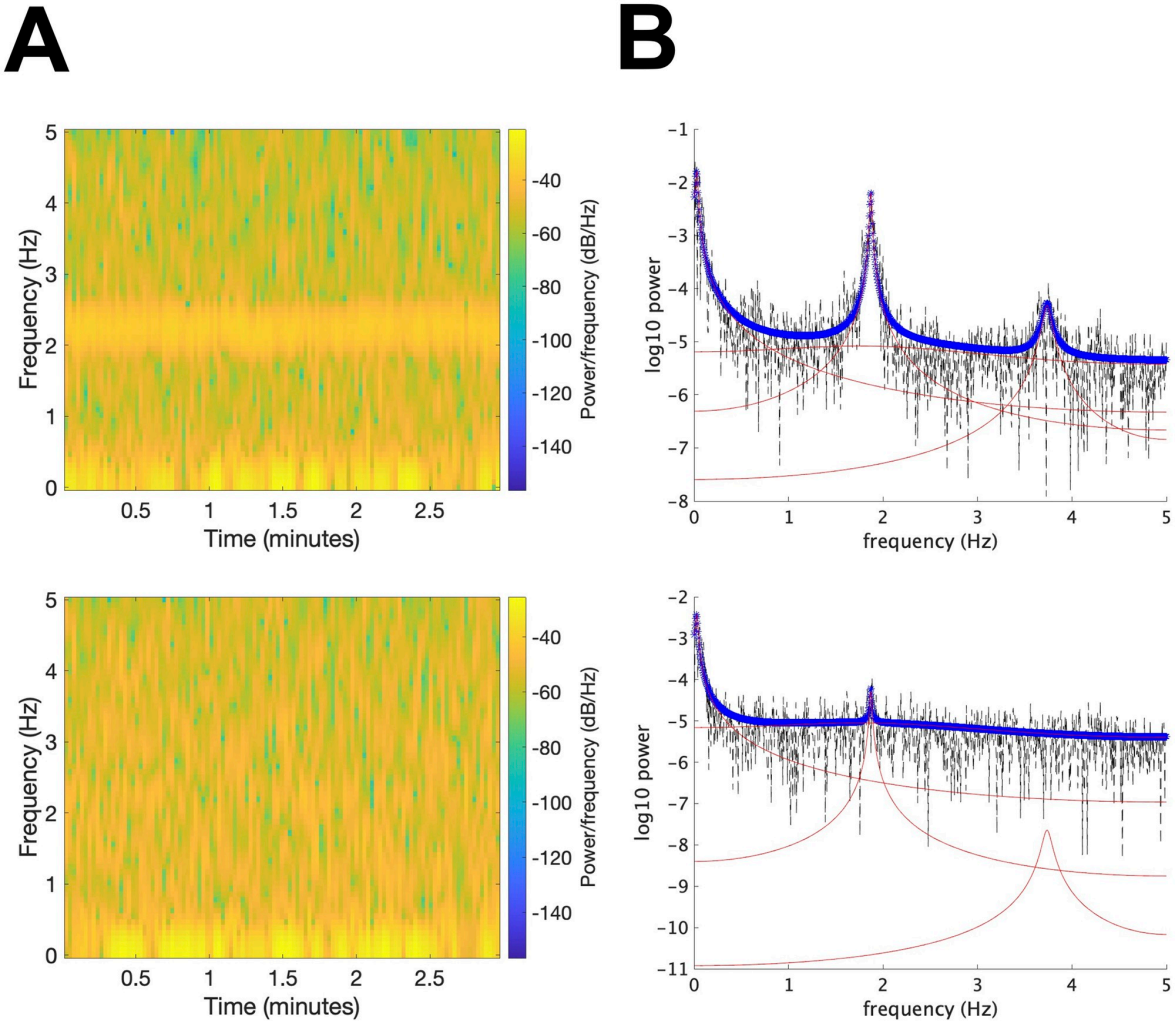
(A) Spectrogram of infant fNIRS data in Fig 8. Upper: oxy-Hb; lower: deoxy-Hb. (B) Periodogram (black), spectral density functions of the fitted oscillators (red) and their sum (blue) for infant fNIRS data in Fig 8. Upper: oxy-Hb; lower: deoxy-Hb.

Fig 7 presents one example, in which four oscillators with frequencies 0.028, 1.84, 1.87, 3.74 Hz are extracted. The results are shown for the first 20 seconds. The first oscillator is considered to reflect hemodynamic changes in response to brain activity, whereas the second and third oscillators are interpreted as mirroring noise and cardiac pulse waves, respectively, as will be explained below. The second oscillator superposes on oxy- and deoxy-Hb with almost the same power, whereas the third and fourth oscillators superpose less on deoxy-Hb compared to oxy-Hb. Given that the frequency of the fourth oscillator is twice that of the third oscillator, the fourth oscillator is considered to be the harmonic of the third oscillator. Fig 8 shows the results for the entire 3 minutes and Fig 9 shows the estimated projection pattern. Fig 10 shows the spectrogram and specta. In Fig 10B, in addition to the periodogram of the raw data, the spectral density functions of the fitted oscillators (ARMA(2,1) model) and their sum are also plotted. See [16] for mathematical details. For oxy-Hb, both spectrogram and periodogram have two peaks around 0 Hz and 2 Hz, which correspond well to the first and second/third oscillators, respectively. For deoxy-Hb, only the peak around 0 Hz is clear. Thus, the spectrogram and spectra do not provide enough information to extract common oscillators underlying oxy- and deoxy-Hb time series. In particular, it is difficult to figure out that there are two types of oscillators with different projection patterns around 2 Hz by visual inspection. OSC-DECOMP can separate these oscillators in a data-driven manner by statistical model fitting and model selection with AIC.

**Fig 11:**
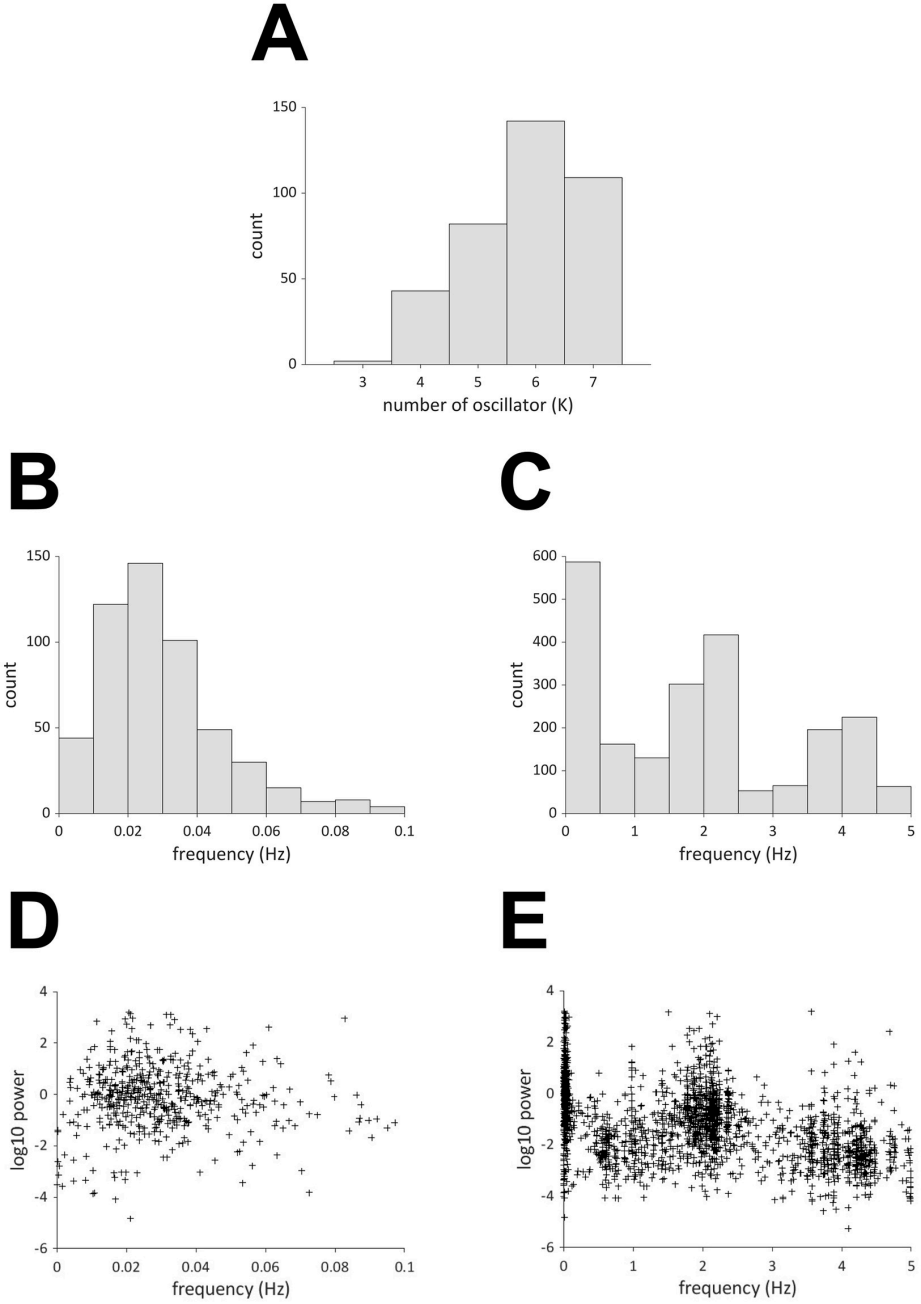
(A) Histogram of the number of extracted oscillators. (B) (C) Histogram of the frequency of extracted oscillators. (D) (E) Scatter plot of the frequency and power of the extracted oscillators.

Fig 11 summarizes the properties of the extracted oscillators for the 21 infants and 18 channels. Fig 11A presents a histogram of the number of extracted oscillators. Five to seven oscillators were extracted in most cases. Fig 11B and 11C shows a histogram of the frequency of the extracted oscillators. There are three peaks near 0.01–0.1 Hz, 1.6–2.4 Hz, and 3.6–4.4 Hz. The first peak is considered to reflect brain activity, whereas the second peak is interpreted as cardiac pulse waves or mirroring noise, as will be explained below. The third peak is regarded as the harmonic of the second peak. Fig 11D and 11E shows scatter plots of the frequency and power of the extracted oscillators. The power tends to be smaller for higher frequencies.

**Fig 12:**
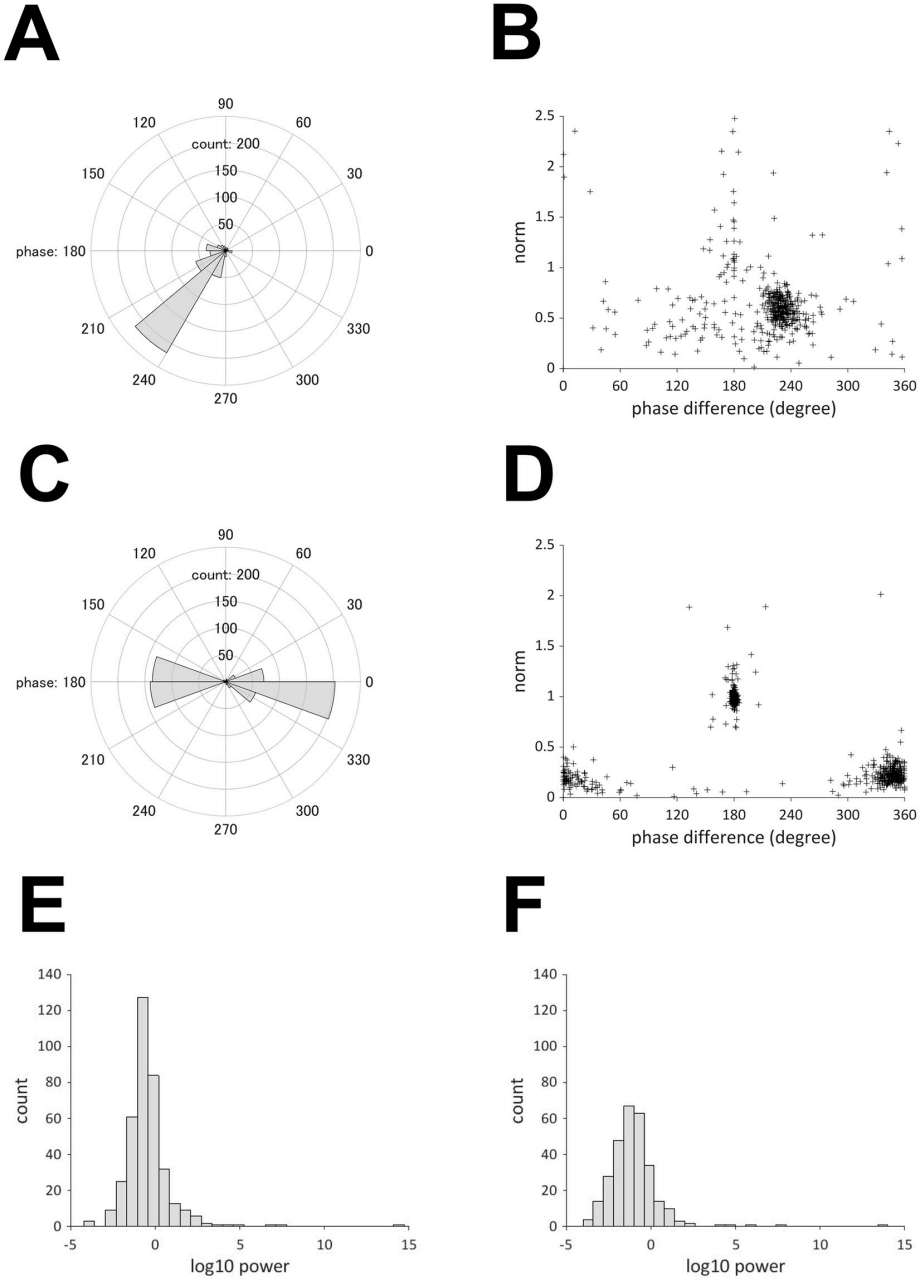
(A) Histogram of phase difference (0.01–0.1 Hz). (B) Scatter plot of phase difference and norm of the projection vector (0.01–0.1 Hz). (C) Histogram of phase difference (1.6–2.4 Hz). (D) Scatter plot of phase difference and norm of projection vector (1.6–2.4 Hz). (E) Histogram of power of pulse wave. (F) Histogram of the power of the mirroring noise.

Fig 12 presents the projection pattern of the oscillators with frequencies of 0.01–0.1 Hz or 1.6–2.4 Hz. For oscillators with a frequency of 0.01–0.1 Hz, the phase difference between oxy- and deoxy-Hb is approximately 230 degrees (Fig 12A), which is consistent with the findings by [14]. The norm of the projection vector on deoxy-Hb is distributed in the range of 0.5 to 0.7 (Fig 12B), which indicates that these oscillators superpose less on deoxy-Hb compared to oxy-Hb. Since the slow oscillations between 0.01 Hz and 0.1 Hz are likely to reflect hemodynamic changes in response to the spontaneous activity of the brain, they are referred to as “brain oscillators.” Regarding the oscillators with a frequency range of 1.6–2.4 Hz, which coincide with the frequency of cardiac pulses, the phase difference has two peaks at 0 degrees and 180 degrees (Fig 12C). Fig 12D shows that the norm of the projection vector on deoxy-Hb is approximately 0.2 for oscillators with a phase difference of approximately 0 degrees, whereas it is approximately one for oscillators with a phase difference of approximately 180 degrees. Therefore, there are two types of oscillators in the frequency band of 1.6–2.4 Hz. The first type is superposed on oxy- and deoxy-Hb with almost the same phase, and its effect on oxy-Hb is approximately five times larger than that on deoxy-Hb. The second type is superposed on oxy- and deoxy-Hb with almost the same power and opposite phase. The in-phase changes in oxy- and deoxy-Hb can be generated by cardiac pulses because the associated oscillatory changes in the blood volume produce simultaneous changes in both oxy- and deoxy-Hb. In contrast, the anti-phase changes in oxy- and deoxy-Hb can be generated by measurement noise. According to the Lambert–Beer law [29], oxy- and deoxy-Hb changes are calculated as

$$\begin{array}{c} \Delta \text{OD}\left(\lambda \right)=(\varepsilon_{o}\left(\lambda \right)\Delta C_{o}^{e}+\varepsilon_{d}\left(\lambda \right)\Delta C_{d}^{e})L, \end{array}$$

where ΔOD(λ) is the change in optical density measured at a given wavelength λ, $\Delta C_{o}^{e}$ and $\Delta C_{d}^{e}$ are estimated changes in concentration of oxy- and deoxy-Hb, respectively, *ε*_*o*_(λ) and *ε*_*d*_(λ) are the extinction coefficients of oxy- and deoxy-Hb at a given wave length λ, respectively, and *L* is the optical pathlength. However, the detected intensities of the two wavelengths of light include measurement noise in addition to changes due to absorption by chromophores as

$$\begin{array}{c} \Delta \text{OD}\left(\lambda \right)=(\varepsilon_{o}\left(\lambda \right)\Delta C_{o}^{r}+\varepsilon_{d}\left(\lambda \right)\Delta C_{d}^{r})L+N\left(\lambda \right), \end{array}$$

where $\Delta C_{o}^{r}$ and $\Delta C_{d}^{r}$ are real changes in concentration of oxy- and deoxy-Hb, respectively, and *N*(λ) is applied noise at a given wave length λ. Then, we obtain the following equation as

$$\begin{array}{c} \varepsilon_{o}\left(\lambda \right)\Delta C_{o}^{e}+\varepsilon_{d}\left(\lambda \right)\Delta C_{d}^{e}=\varepsilon_{o}\left(\lambda \right)\Delta C_{o}^{r}+\varepsilon_{d}\left(\lambda \right)\Delta C_{d}^{r}+N\left(\lambda \right)/L. \end{array}$$

For measurements of ΔOD(λ) at two wavelengths the above equation are given by

$$\begin{array}{c} \varepsilon_{o}\left(\lambda_{1}\right)\Delta C_{o}^{e}+\varepsilon_{d}\left(\lambda_{1}\right)\Delta C_{d}^{e}=\varepsilon_{o}\left(\lambda_{1}\right)\Delta C_{o}^{r}+\varepsilon_{d}\left(\lambda_{1}\right)\Delta C_{d}^{r}+N\left(\lambda_{1}\right)/L, \end{array}$$

$$\begin{array}{c} \varepsilon_{o}\left(\lambda_{2}\right)\Delta C_{o}^{e}+\varepsilon_{d}\left(\lambda_{2}\right)\Delta C_{d}^{e}=\varepsilon_{o}\left(\lambda_{2}\right)\Delta C_{o}^{r}+\varepsilon_{d}\left(\lambda_{2}\right)\Delta C_{d}^{r}+N\left(\lambda_{2}\right)/L. \end{array}$$

**Fig 13:**
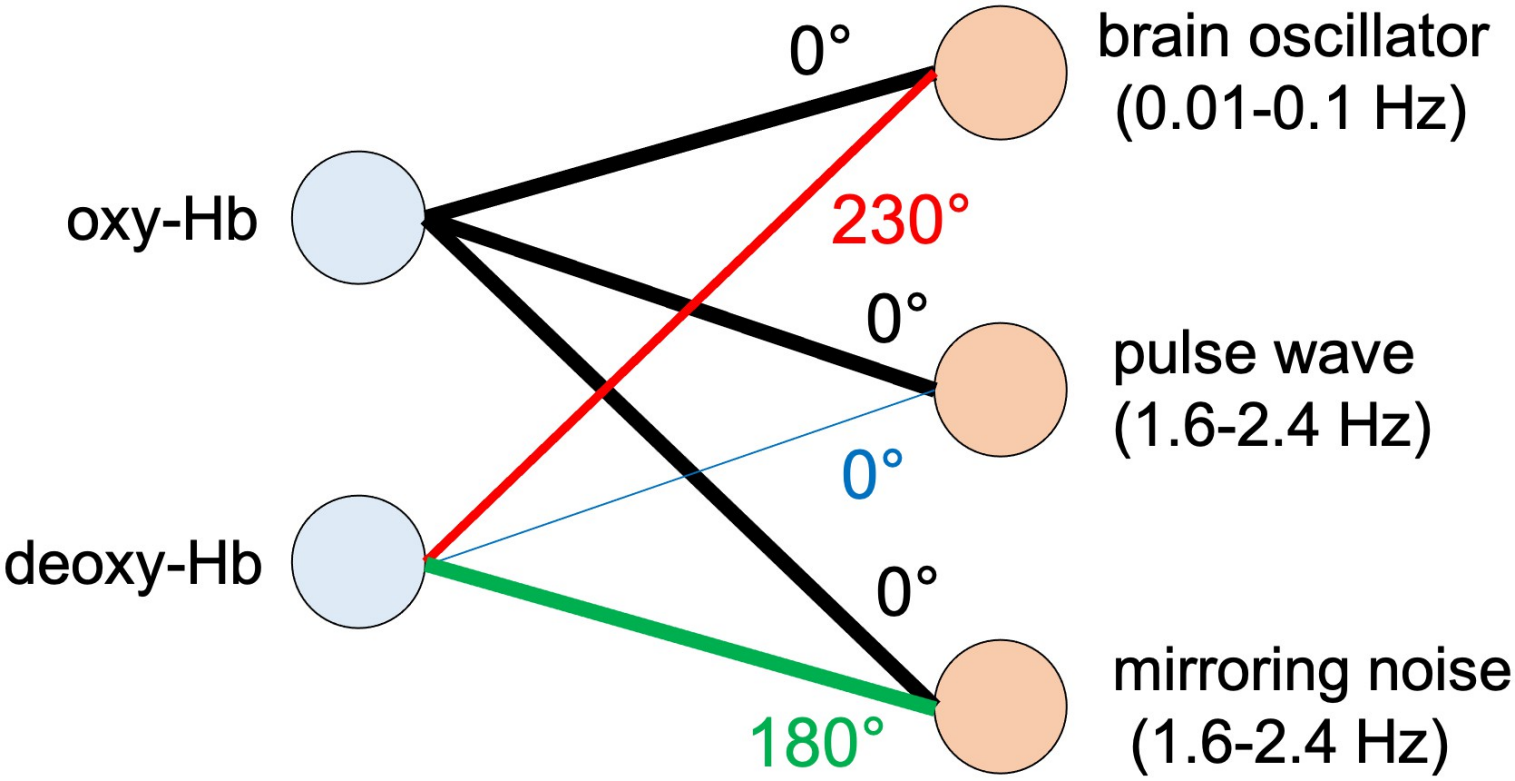
Projection pattern of three types of oscillators in infant fNIRS.

A few lines of algebra indicates that

$$\begin{array}{c} \Delta C_{o}^{e}=\Delta C_{o}^{r}-E\left(\varepsilon_{d}\left(\lambda_{2}\right)N\left(\lambda_{1}\right)-\varepsilon_{d}\left(\lambda_{1}\right)N\left(\lambda_{2}\right)\right), \end{array}$$

$$\begin{array}{c} \Delta C_{d}^{e}=\Delta C_{d}^{r}+E\left(\varepsilon_{o}\left(\lambda_{2}\right)N\left(\lambda_{1}\right)-\varepsilon_{o}\left(\lambda_{1}\right)N\left(\lambda_{2}\right)\right), \end{array}$$

where *E* = 1/(*ε*_*o*_(λ_2_)*ε*_*d*_(λ_1_) − *ε*_*o*_(λ_1_)*ε*_*d*_(λ_2_))*L*. This shows that the measurement noise for each wavelength is transformed to noisy signals of oxy- and deoxy-Hb with opposite sign. Thus, the first type is referred to as a “pulse wave oscillator,” and the second type as a “mirroring noise oscillator.” Fig 12E and 12F presents the histogram of the power of these two types of oscillators. Fig 13 summarizes these findings.

**Fig 14:**
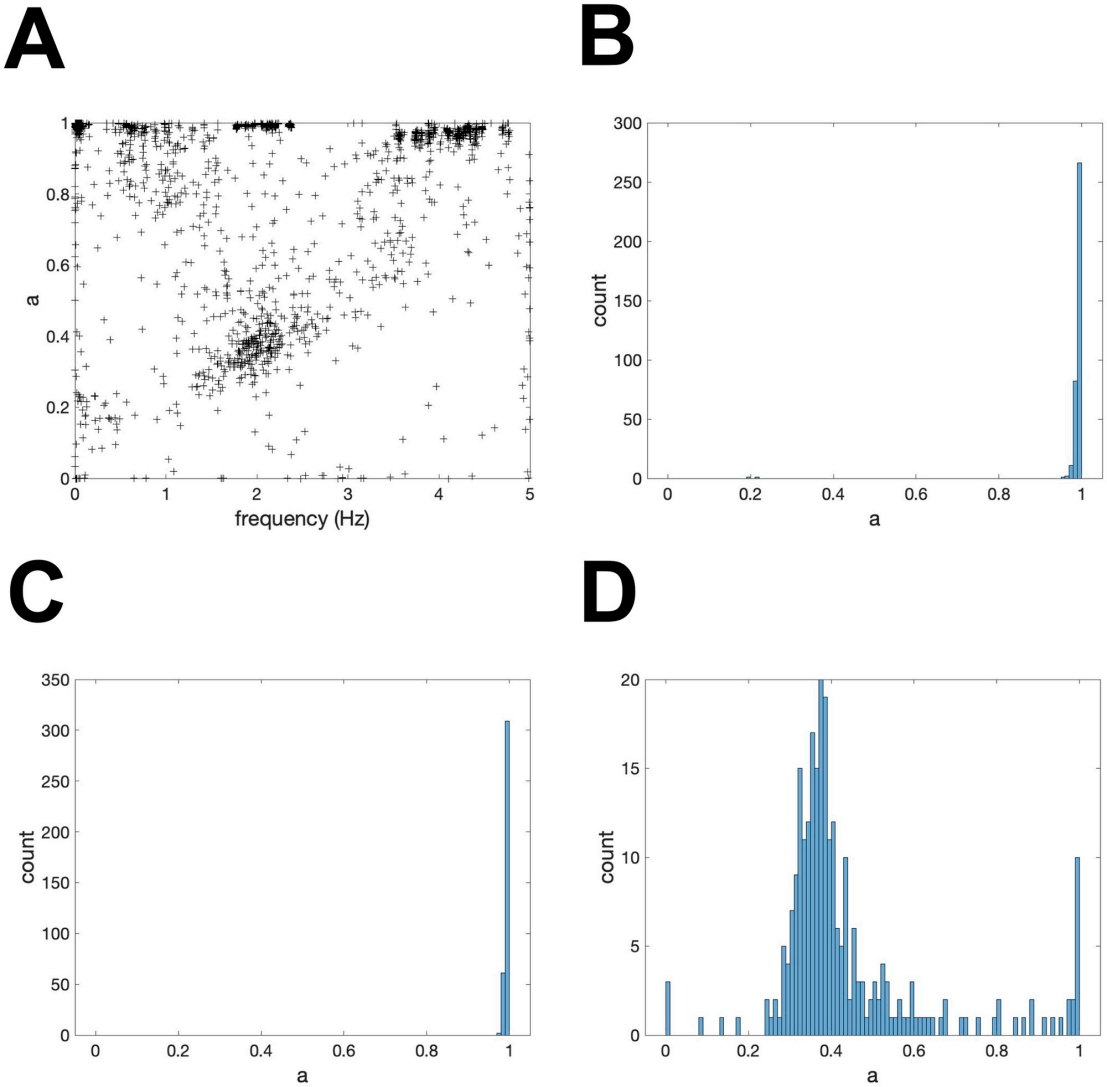
(A) Scatter plot of the frequency *f*_*k*_ and parameter *a*_*k*_ of the extracted oscillators. (B) Histogram of the parameter *a*_*k*_ for the brain oscillators. (C) Histogram of the parameter *a*_*k*_ for the pulse wave oscillators. (D) Histogram of the parameter *a*_*k*_ for the mirroring noise oscillators.

In the oscillator model (1), the parameter *a*_*k*_ ∈ (0, 1) specifies the width of the spectral peak at the frequency *f*_*k*_. Namely, the width of the spectral peak is smaller for *a*_*k*_ closer to one. Fig 14 shows the distribution of *a*_*k*_ for each type of oscillators. It indicates that the spectral peak is very sharp for the brain oscillators and pulse wave oscillators. In other words, the waveform is close to sinusoidal for these oscillators. It implies that the brain oscillators cannot be interpreted as non-oscillatory drift, although its frequency is close to zero. On the other hand, for the mirroring noise oscillators, the parameter *a*_*k*_ is concentrated around 0.4. Thus, the mirroring noise is considered to be non-sinusoidal.

### Testing of common oscillator hypothesis

**Fig 15:**
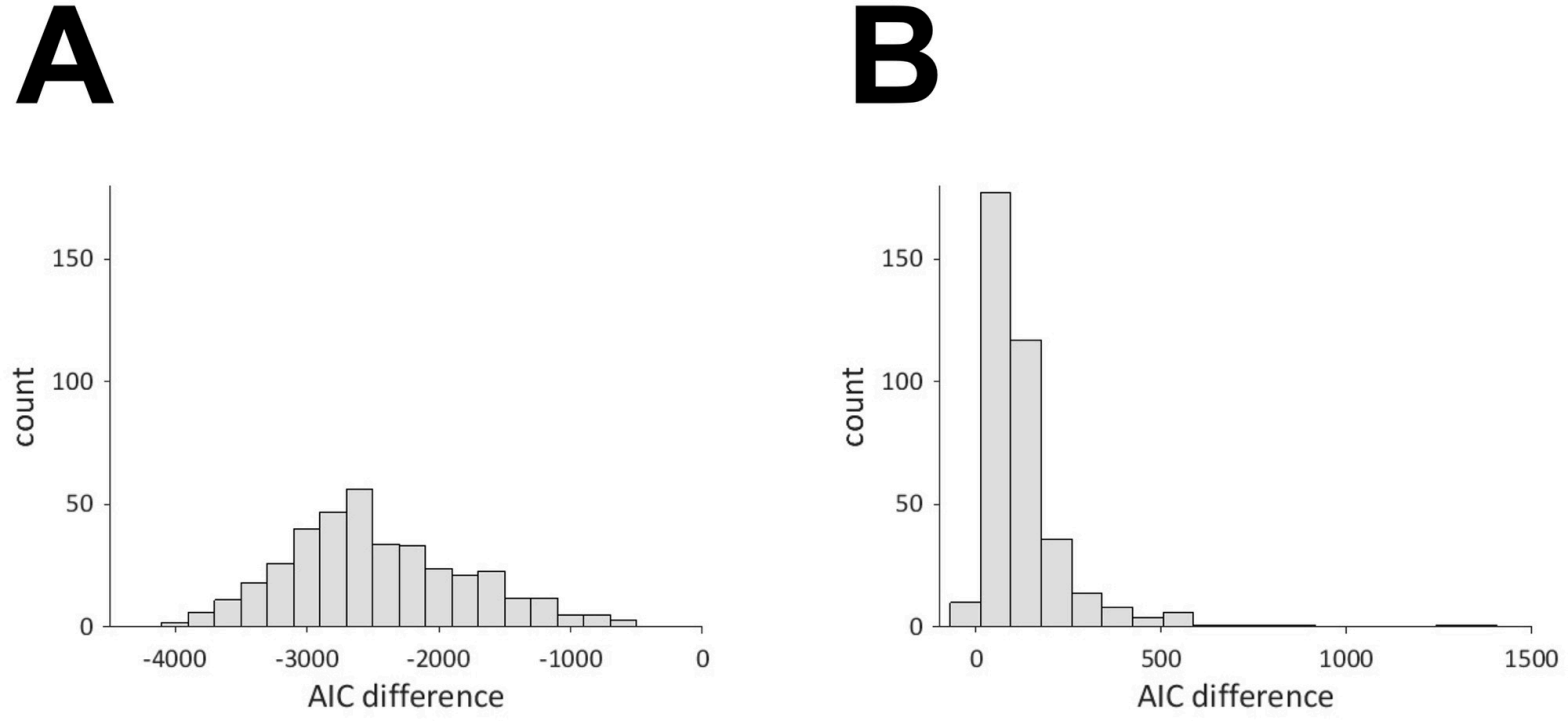
Histogram of AIC difference for (A) same channel and (B) different channels.

To verify that the time series of oxy- and deoxy-Hb on each channel originate from common oscillatory activity, we compared the Akaike Information Criterion (AIC) of two state space models. Fig 15A shows the histogram of the AIC difference, wherein we have 378 data points corresponding to the 18 channels of 21 infants. This shows that the AIC is smaller for the model with common oscillators in all cases. By using the extension of Linhart’s test (see Material and methods), these AIC differences were found to be significant with *p* < 10^−16^ in all cases. The Wilcoxon signed-rank test, which does not require the normality assumption on the test statistic, also demonstrated that these AIC differences were significant with *p* < 10^−12^ in more than 90% cases. Therefore, the assumption that common oscillators underlie the oxy- and deoxy-Hb time series is indeed supported by the experimental data. For comparison, we applied the same analysis to pairs of oxy- and deoxy-Hb data that were randomly shuffled across the subjects. The result is shown in Fig 15B. In this case, the AIC is larger for the model with common oscillators in all cases except for three.

### Frequency-specific functional connectivity

**Fig 16:**
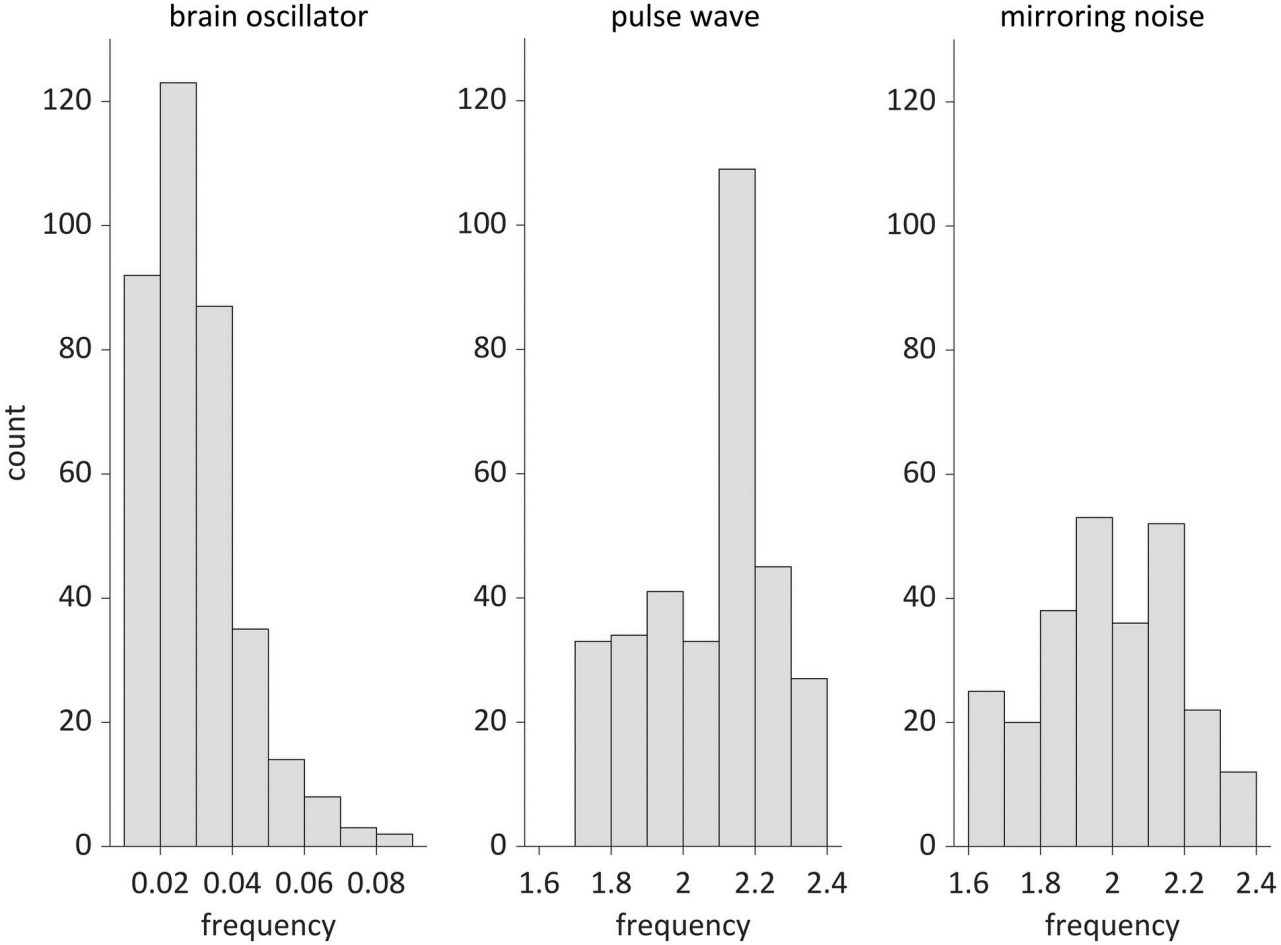
Histogram of the frequency of the oscillators used in the functional connectivity analysis.

**Fig 17:**
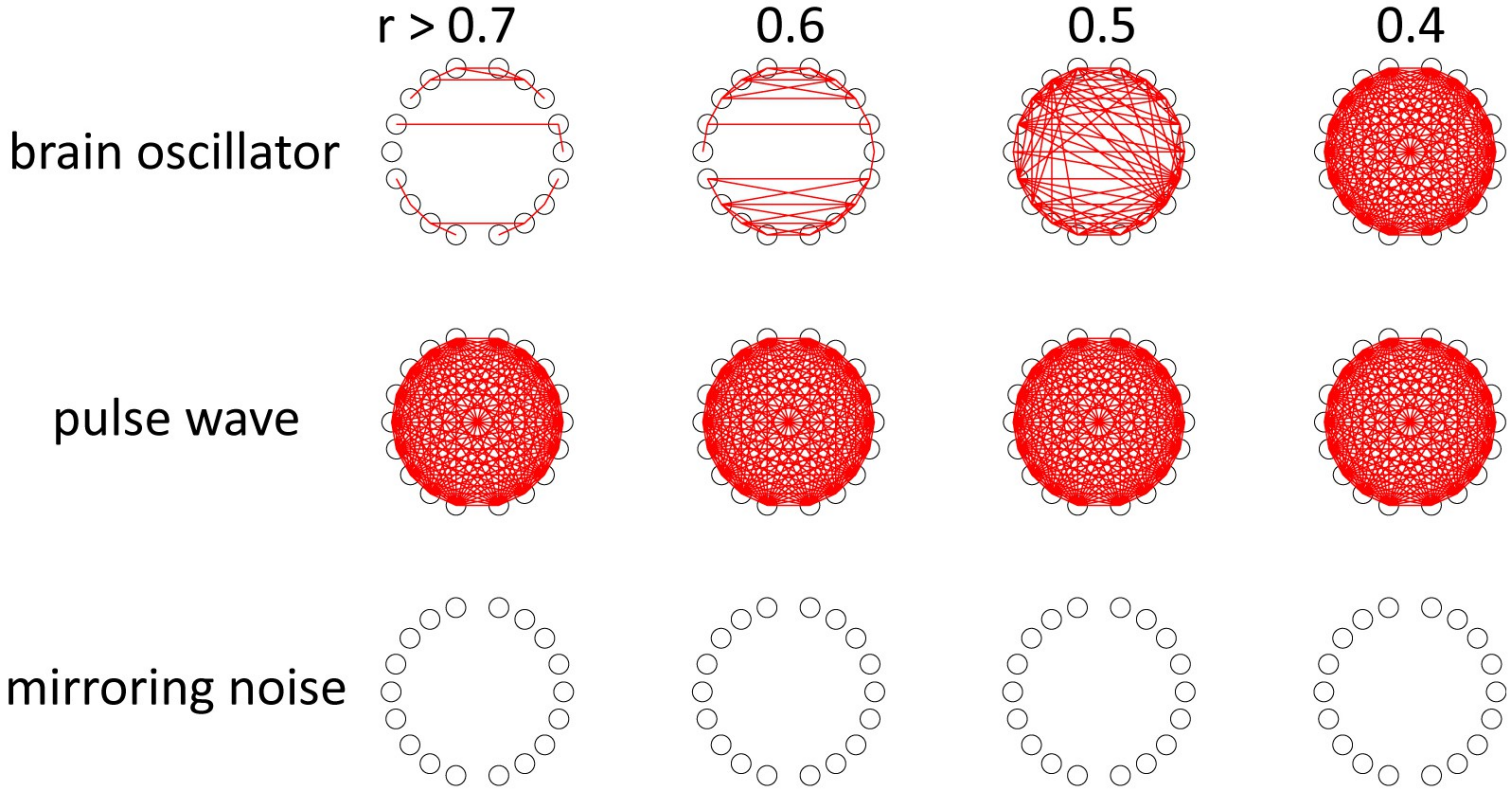
Frequency-specific functional connectivity or spatial synchrony. The numbers at the top represent the correlation coefficients of the thresholds. Upper: 0.01–0.1 Hz, middle: 1.6–2.4 Hz (in-phase), lower: 1.6–2.4 Hz (antiphase).

Finally, we examined the frequency-specific functional connectivity using the canonical correlation coefficients as explained in Material and Methods. We considered that this method revealed spatial synchrony when oscillators of pulse waves and mirroring noise were analysed. For each of the three oscillators shown in Fig 13, we focused on the oscillator with the highest power. The average number of infants with oscillators was as follows: brain oscillators = 20.2 per channel, pulse wave = 20.7 per channel, and mirroring noise = 14.6 per channel. The number of infants with mirroring noise was lower compared to the other two oscillators because this noise was not always mixed with the fNIRS signal. Fig 16 shows the frequency distribution of the oscillators. For brain oscillators (0.01–0.1 Hz), oscillators with frequencies between 0.02 Hz and 0.03 Hz were most often observed, and the number decreased as the frequency increased (mean: 0.029 Hz, mean frequency range between 18 channels: 0.024–0.037 Hz). Although the oscillators of the mirroring noise (1.6–2.4 Hz, antiphase) exhibited frequencies between 2.1 Hz and 2.2 Hz (mean: 2.075 Hz, mean frequency range: 2.068–2.084 Hz), the distribution of the pulse waves did not have such a peak (mean: 1.993 Hz, mean frequency range: 1.853–2.074 Hz). Friedman’s tests for the brain oscillators did not show significant differences in the frequencies between the channels after Bonferroni correction was performed for multiple comparisons. However, there were significant differences between the two-channel pairs in the tests for pulse waves (L5 and L9, *p* < 0.05; L5 and R8, *p* = 0.005) and mirroring noise (L3 and R3: *p* < 0.05; L9 and R3: *p* < 0.05). Fig 17 shows the average of the canonical correlation coefficients for the 21 infants. The results for the brain oscillators are presented in Fig 17 (upper). When the threshold is set to 0.7 or 0.6, there are primarily two types of connections: homologous connectivity (inter-hemispheric connectivity between homologous regions) and short-distance ipsilateral connectivity. In addition, when the threshold was set to 0.5, long-distance ipsilateral connectivity between the frontal and temporal/occipital regions was also found. These results are similar to previously reported findings for adult participants [30]. Fig 17 (middle) shows the results for pulse wave oscillators. The correlation is much larger regardless of the channel locations, and most channels are connected even when the threshold is set to 0.7. Fig 17 (lower) shows the results for the mirroring noise oscillators. The correlation between the channels is extremely small, and no connection appears even when the threshold is set to 0.4.

**Fig 18:**
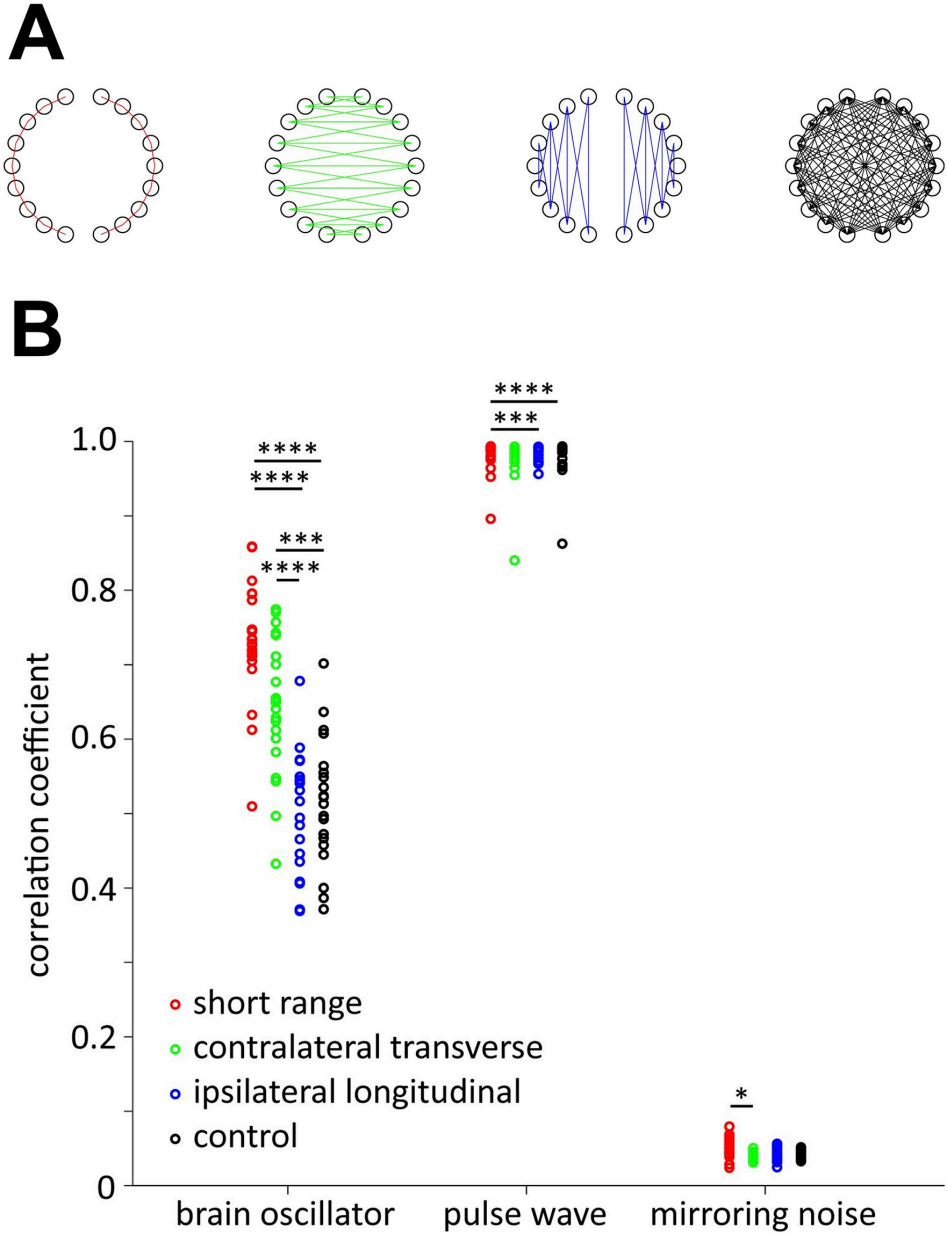
Mean correlation coefficients of each infant for the four types of functional connectivities. (A) Short range (red), contralateral transverse (green), ipsilateral longitudinal (blue), and control (black) connectivities. (B) Red, green, blue, and black lines indicate individual data for short-range, contralateral transverse, ipsilateral longitudinal, and control connectivity, respectively. **** *p* < 0.001, *** *p* < 0.005, ** *p* < 0.01, and * *p* < 0.05 after Bonferroni correction for multiple comparisons.

To evaluate the aforementioned visual inspections, we categorized channel pairs into the following four groups (Fig 18A) based on a previous study [31]: (i) short-range connectivity (16 pairs), (ii) contralateral-transverse connectivity (25 pairs), (iii) ipsilateral-longitudinal connectivity (20 pairs), and (iv) control (92 pairs). Control connectivity consisted of pairs other than (i), (ii), and (iii). Fig 18B shows averaged values of coefficients within each group. The averaged values of the pulse waves and those of the mirroring noise were the highest and the lowest, respectively, and the values of the brain oscillators were intermediate. They were mostly higher than 0.4. This result indicates that the pulse wave is superposed on most channels with similar frequencies and phases, but the presence of mirroring noise in the signal depends on the measurement channel. Regarding brain oscillators, Friedman’s test revealed that short-range and contralateral-transverse connectivities were significantly larger than ipsilateral-longitudinal and control connectivities, which support the aforementioned assertions. The threshold of 0.7 in Fig 17, represents short-range connectivity; the threshold of 0.6 exhibits short-range and contralateral-transverse connectivities; the threshold of 0.5, which shows short-range, contralateral-transverse, and a part of the ipsilateral-longitudinal and control connectivities, and the threshold of 0.4 exhibits most connectivities.

**Fig 19:**
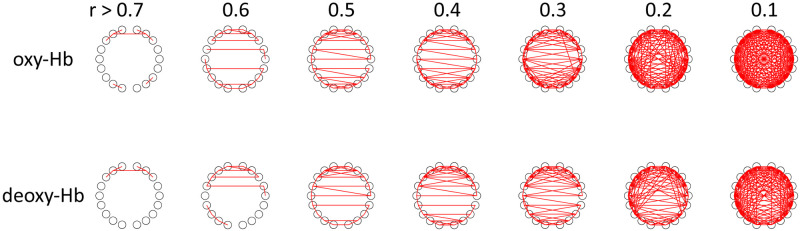
Functional connectivity in the frequency range 0.01–0.1 Hz computed by correlation coefficients. The numbers at the top represent the correlation coefficients of the thresholds. Upper: oxy-Hb, lower: deoxy-Hb.

For comparison, Fig 19 shows the functional connectivity computed by the correlation coefficients for oxy-Hb and deoxy-Hb in the frequency range 0.01–0.1 Hz. For both oxy-Hb and deoxy-Hb, short-range and contralateral connectivities are stronger than other types of connectivities. Thus, the network structure is similar to the functional connectivity of brain oscillators in Fig 17. The value of the correlation coefficients is larger in oxy-Hb than deoxy-Hb, which is compatible to previous studies. They tend to take smaller values than the canonical correlation coefficients shown in Fig 17. It may be due to the denoising effect of OSC-DECOMP. It is an interesting future work to explore this more.

### Comparison with empirical mode decomposition

Here, we compare OSC-DECOMP with the empirical mode decomposition (EMD) [32]. EMD is a method for analyzing nonlinear and nonstationary time series data by decomposing them into several oscillatory components called the “intrinsic mode functions.” By applying the Hilbert transform, the instantaneous frequency of each intrinsic mode function is computed. Thus, the time-frequency distribution of signal amplitude is obtained and it is called the Hilbert spectrum or Hilbert–Huang transform.

**Fig 20:**
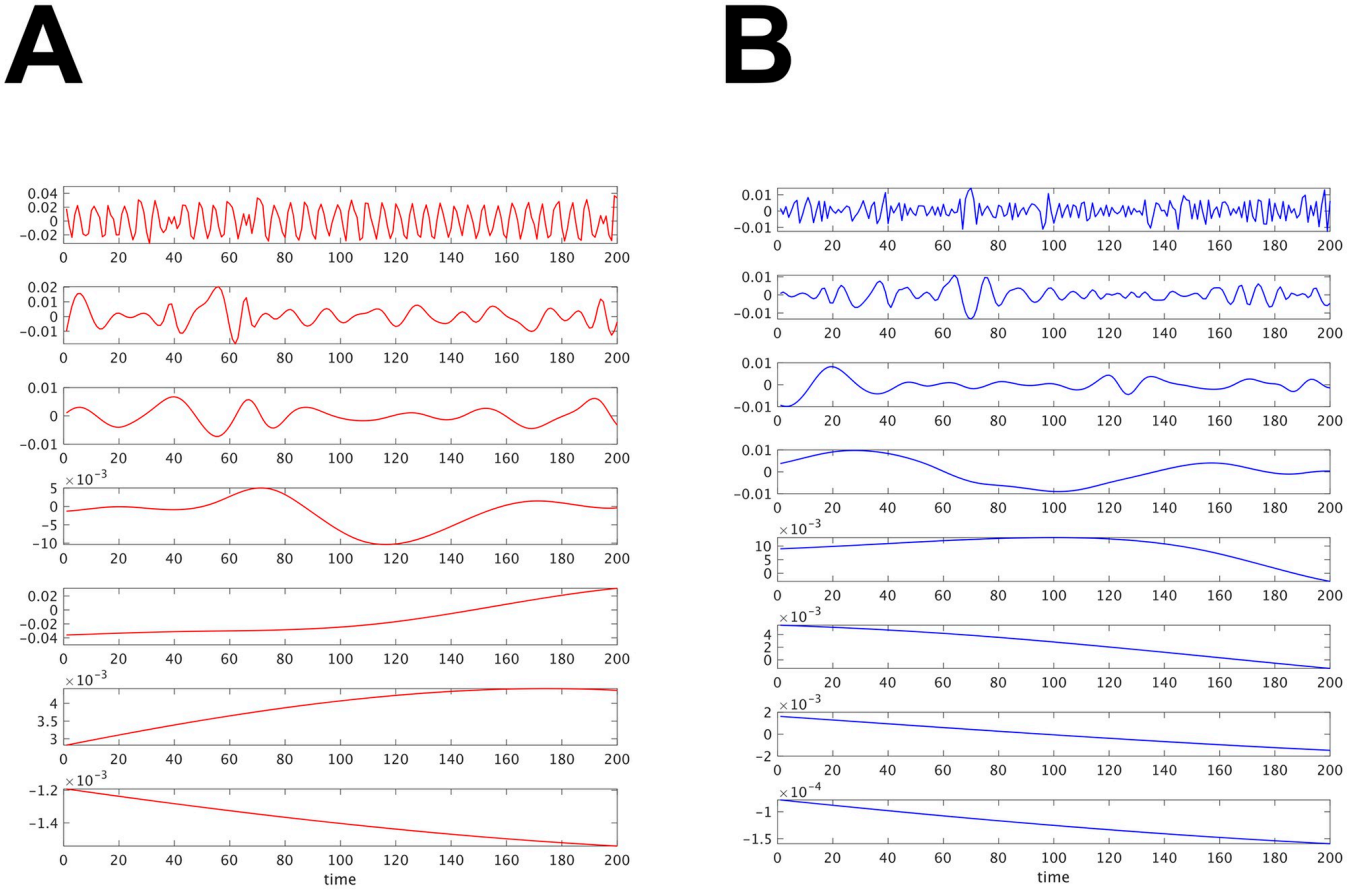
(A) Intrinsic mode functions in the oxy-Hb. (B) Intrinsic mode functions in the deoxy-Hb.

Fig 20 shows the intrinsic mode functions (IMF) obtained by EMD (MATLAB function “emd”) for the infant fNIRS data in Fig 7A. In this case, EMD detected seven and eight components in oxy-Hb and deoxy-Hb, respectively. The first IMF seems to correspond to the pulse wave oscillator. The other IMFs have lower frequencies.

EMD is an algorithm for decomposing univariate nonstationary time series data into several mode functions by focusing on the local properties of the signal. It does not aim at extracting common oscillators in multivariate time series data. On the other hand, OSC-DECOMP extracts oscillators from both univariate and multivariate stationary time series data based on statistical models. It is an interesting future work to extend OSC-DECOMP to nonstationary data.

## Discussion

In this study, we investigated the oscillatory activity in infant fNIRS data by using the oscillator decompisition method (OSC-DECOMP) [16, 17]. Five to seven oscillators were extracted on most channels and their frequency distribution had three peaks at 0.01–0.1 Hz, 1.6–2.4 Hz and 3.6–4.4 Hz. The first peak was considered to reflect brain activity and the phase difference between oxy- and deoxy-Hb for the oscillators were at approximately 230 degrees. The second peak involved two types of in-phase and anti-phase oscillations of oxy- and deoxy-Hb. The former and latter are attributed to cardiac pulse waves and mirroring noise. The third peak was regarded as the harmonic of the second peak. By comparing the Akaike Information Criterion (AIC) of the two state space models, we verified that the time series of oxy- and deoxy-Hb on each channel originate from common oscillatory activity. We also applied the results of OSC-DECOMP to investigate the frequency-specific functional connectivity.

### Advantages of OSC-DECOMP

Neural oscillations are relevant in many brain functions [33]. For example, the electroencephalogram (EEG) time series is composed of several oscillation components (e.g., alpha, beta, and gamma), and each oscillation component has its physiological role. Conventionally, neural oscillation analysis is conducted by applying band-pass filters to the time series data [34]. The outputs of the band-pass filters are assumed to represent the target oscillation components. For example, the output of a filter with a pass-band of 8–13 Hz is considered as the alpha component. Although such conventional methods are widely used and easy to implement, there are several associated limitations [35]. In particular, the band-pass filter is often selected arbitrarily, and this affects the final result. For example, even the definition of the alpha band seems to vary among studies, such as 8–13 Hz and 9–12 Hz.

In contrast, OSC-DECOMP extracts the oscillation components in a data-driven (objective) manner using a statistical modeling approach. Both the number and frequencies of underlying oscillators are determined based on data. When applied to multivariate time series data, this method also estimates the projection pattern between each time series and each oscillator, which describes the amplitude and phase modulation. Thus, even if there are oscillators with very close frequencies, OSC-DECOMP can separate them correctly if their projection patterns are sufficiently different. The current result on pulse wave and mirroring noise can be interpreted as a typical example of this. Therefore, OSC-DECOMP enables the investigation of the common oscillators that underly multivariate time series in a data-driven manner. Several studies have focused on the phase difference between the band-pass filtered signals of oxy- and deoxy-Hb. For example, [14] investigated the phase difference at a frequency of 0.05–0.1 Hz in infant fNIRS data and discussed its relationship with preterm birth. Our findings may provide a foundation for such investigations of phase differences. It should also be noted that the framework of state-space models naturally leads to real-time phase estimation [36].

### Oscillators underlying infant fNIRS data

The present study based on OSC-DECOMP demonstrated that the brain, pulse wave, and mirroring noise oscillators can be extracted from the fNIRS signals obtained from the cranium of sleeping infants. In the time domain, fNIRS signals with slow components between 0.01 Hz and 0.1 Hz generally include hemodynamic and oxygenation changes in response to brain activity, similarly to fMRI [37–39]. The present study revealed that oscillators with frequencies between 0.02 Hz and 0.03 Hz were most common, as shown in Fig 11B and 11D. Furthermore, the phase difference between oxy- and deoxy-Hb chambers was approximately 230 degrees, as shown in Fig 12A and 12B, which reflects complex mechanisms for hemodynamics and oxygenation [14]. This method can serve as a new approach for studying brain activation and connectivity using the decomposed oscillators of fNIRS signals. In addition to the changes induced by brain activity, the slow components of fNIRS signals are subject to systemic influences such as heart rate and blood pressure [40]. Although it was not proven that the brain oscillator was purely neurogenic in the present study, data-driven decomposition of the oscillator will simplify the procedure for determining if some oscillation components are associated with systemic oscillations.

For frequencies higher than 0.1 Hz, fNIRS signals include components associated with respiration (-0.3 Hz) and heartbeat (-1 Hz), in the case of adults [41]. In the present study, although distinct oscillators corresponding to respiration were not observed, the conspicuous oscillators caused by cardiac pulse waves were extracted at approximately 2 Hz, as shown in Fig 11C and 11E. The oscillators included in-phase and anti-phase oscillations of oxy- and deoxy-Hb, as shown in Fig 12C and 12D. The in-phase changes in oxy- and deoxy-Hb data can be attributed to blood volume changes induced by pulse waves [11, 42]. The anti-phase changes in oxy- and deoxy-Hb can be attributed to mirroring noise that is artificially produced during the calculation of the concentration of two chromophores using two-wavelength absorption based on the Lambert-Beer equation [29]. The measurement noise in the intensities of the detected light at each wavelength is transformed to noisy signals of oxy- and deoxy-Hb with opposite signs. The mirroring changes in oxy- and deoxy-Hb can also be caused by insufficient separation of chromophore changes due to the wavelength dependence of the light path length [43, 44]. As we assumed the same optical path length for the two wavelengths, the estimated signals associated with oxy- and deoy-Hb may have cross-talk. This cross-talk is not confined to the higher frequency range, but can also occur in the lower frequency range. Moreover, a small number of oscillators with a phase difference of 180° was observed in the slower frequency range, as shown in Fig 12B.

fNIRS signals are assumed to reflect changes in hemodynamics and oxygenation in cerebral tissue. However, those that originate from extracerebral tissue can have an impact on the fNIRS signals [45]. In the present study, we used data acquired from sleeping 3-month-old infants using a source-detector distance of 2 cm. A previous study involving infants of the same age showed that 2 cm is the optimal inter-optode distance for the detection of hemodynamic changes in response to auditory stimulation [46]. Based on the deep-shallow signal separation method using multi-distant probes and independent component analysis, it was further revealed that the deep tissue contribution during sleeping with a 2 cm separation was 66–79% for oxy-Hb changes [47]. Thus, the brain oscillator in the present study primarily reflects the hemodynamics and oxygenation of cerebral tissue. To gain more detailed information exclusive to brain activation by combining decomposition methods of fNIRS signals in the time domain of oscillations and in the spatial domain of tissue depth, further studies are required.

### Frequency-specific functional connectivity in fNIRS and future studies

In this study, we examined frequency-specific functional connectivity by calculating the canonical correlation between two oscillators in different channels (Figs 17 and 18). We found that each of the three types of oscillators (i.e., brain oscillators, pulse waves, and mirroring noise) showed individual patterns of functional connectivity or spatial synchrony. Conventionally, oscillator analysis and functional connectivity of fNIRS signals utilize the coherence between band-pass filtered signals [3, 10, 13, 30, 48]. For example, [30] revealed that the connectivity pattern depends on the frequency band, and short-range and long-range connectivities are manifested in oxy-Hb signals at frequencies below 0.1 Hz. It would be interesting to investigate the relationship between the proposed oscillator-based method and the conventional coherence-based method. The most obvious difference between the two methods is the processing of the two types of Hb signals. The conventional method treats the two types of Hb signals separately, but the proposed method can treat them individually or together. The deoxy-Hb signals were smaller than oxy-Hb signals, and the measurement noise had a greater effect on the coherence between the channels in the deoxy-Hb signals compared to the oxy-Hb signals. As a result, there was a tendency to rely on oxy-Hb signals when summarizing the results obtained using the conventional method. Given that the present method uses common oscillators for the oxy-Hb and deoxy-Hb signals, it has the advantage that the relationship between the channels can be investigated without limiting the oxy-Hb signals. Moreover, Welch’s average periodogram method is often used for calculating coherence, which involves the subjective selection of the window type, window length, and overlap length. In contrast, the proposed method does not require such selections. This reduces the risk that the analysis method will eventually bias the results.

The short-range and contralateral-transverse connectivity of the brain oscillators were higher than the ipsilateral-longitudinal connectivity and the control (Fig 18). These results are consistent with those of previous studies on infants [31] and adults [30]. The short-range and contralateral-transverse connectivity reflect functional relationships based on structural connectivity, such as U-fibers beneath the cortical surface and callosal fibers. Although there are longitudinal fasciculi that connect the anterior and posterior regions, even in infancy [49, 50], the presented and previously reported results consistently showed relatively low synchronization between distant ipsilateral regions. As almost all infants exhibited brain oscillators and the mean frequency of the oscillators was not significantly different between channels, it is possible that the phases of the brain oscillators were not the same in the anteroposterior direction, and the differences caused lower ipsilateral-longitudinal connectivity. This possibility can be examined if we used a longer time series of fNIRS data in a future study.

In the present study, we first determined the brain oscillators for each measurement channel. The functional connectivity was then evaluated by calculating the correlation between the time series of the oscillators. Another way of defining functional connectivity between channels is to determine whether the change in the oxy-Hb and deoxy-Hb signals in two or more channels is caused by common oscillatory activity. For example, each of the two channels measures a set of time series of oxy-Hb and deoxy-Hb signals, and a total of four time series can be used to investigate the presence of a common oscillator. If there is a low-frequency oscillator with power above a certain threshold, these two channels can be considered to exhibit significant functional connectivity. A functional network can also be defined by establishing whether there is a common oscillator among multiple channels. This method can be applied to time series of functional magnetic resonance imaging (fMRI), magnetoencephalography (MEG), electrocorticography (ECoG), and multi-electrode EEG to clarify functional relationships across broad brain regions.

## Supporting information

S1 Appendix Technical appendix. The supplementary material includes the technical details on (1) state space models and time series decomposition, (2) Kalman filter/smoother algorithm, and (3) Hessian computation and confidence interval.
(PDF)
