# Supplementary material for: Oscillator decomposition of infant fNIRS data
Source: PLoS Comput Biol. 2022 Mar 24;18(3):e1009985. doi: 10.1371/journal.pcbi.1009985 (PMC8982875; doi:10.1371/journal.pcbi.1009985)
Supplement: S1 Appendix — The supplementary material includes the technical details on (1) state space models and time series decomposition, (2) Kalman filter/smoother algorithm, and (3) Hessian computation and confidence interval. (PDF) [file pcbi.1009985.s001.pdf]

S1 Appendix: Technical appendix

"Oscillator decomposition of infant fNIRS data"
Takeru Matsuda, Fumitaka Homae, Hama Watanabe, Gentaro Taga, Fumiyasu Komaki

1 State space models and time series decomposition

OSC-DECOMP is based on state space models, which are statistical models of time series data [1, 2]. In state space models, we assume a series of unobserved states $x_1, \dots, x_N$ behind the observed time series data $y_1, \dots, y_N$, where N is the length of the time series. Specifically, a state space model is a pair of the state model

$$x_{t+1} = f(x_t, v_t), \quad v_t \sim p(v), \tag{1}$$

and the observation model

$$y_t = g(x_t, w_t), \quad w_t \sim p(w), \tag{2}$$

where v_t and w_t are called the system noise and the observation noise, respectively. The state model (1) represents the Markov transition of unobserved states, whereas the observation model (2) describes the generation of observed data depending on the current state. In particular, a state space model of the following form is called a Gaussian linear state space model:

$$x_{t+1} = Fx_t + v_t, \quad v_t \sim \mathrm{N}_n(0, Q), \tag{3}$$

$$y_t = Hx_t + w_t, \quad w_t \sim \mathrm{N}_m(0, R), \tag{4}$$

where $x_t \in \mathbb{R}^n$, $y_t \in \mathbb{R}^m$, $F \in \mathbb{R}^{n \times n}$, and $H \in \mathbb{R}^{m \times n}$.

A fundamental task in state space models is to estimate the unobserved states $x = \{x_1, \cdots, x_N\}$ from the observed data $y = \{y_1, \cdots, y_N\}$. We can regard each state variable x_t as a parameter of the distribution $p(y_t \mid x_t)$. The state model (1) is then considered to place a prior on x, and the estimation of x from y in (2) is formulated as a Bayes estimation problem. The posterior distribution $p(x_t \mid y_1, \cdots, y_t)$ and $p(x_s \mid y_1, \cdots, y_t)$ with $s < t$ are called the filtering and smoothing distributions, respectively. For general state space models, several algorithms for filtering and smoothing have been developed, such as the particle filter and the ensemble Kalman filter. In particular, for Gaussian linear state space models, filtering and smoothing are realized using the Kalman filter and Kalman smoother, which require only matrix computations. See [2] for details.

State space models can be used to decompose a given time series into several components. For instance, [3] developed a Bayesian seasonal adjustment method for economic time series. They assumed that a given time series y_t consists of a trend component T_t, seasonal component S_t, and observation noise W_t:

$$y_t = T_t + S_t + W_t.$$

The trend component T_t and seasonal component S_t are described by the Gaussian linear state space models. By applying the Kalman smoother, the given time series y_t is decomposed into these components:

$$y_t = T_{t|N} + S_{t|N} + W_{t|N},$$

where

$$T_{t|N} = E[T_t \mid y_1, \cdots, y_N], \quad S_{t|N} = E[S_t \mid y_1, \cdots, y_N], \quad W_{t|N} = E[W_t \mid y_1, \cdots, y_N].$$

Similarly, [4] proposed a method for decomposing a given time series into trend and AR components.

2 Kalman filter/smoother algorithm

The algorithm of the Kalman filter and smoother is explained in the following. We assume that the initial distribution for x_1 is given by $\mathrm{N}(x_{1|0}, \Sigma_{1|0})$. For example, if the state model (3) is stationary, then we can naturally take the initial distribution as the stationary distribution, which is Gaussian. Then, all of the conditional distributions $p(x_s \mid y_1, \cdots, y_t)$ are Gaussian. Therefore, they are determined by the mean and the covariance matrix. We define the conditional mean as

$$x_{s|t} = E[x_s \mid y_1, \cdots, y_t],$$

and the conditional covariance matrix as

$$\Sigma_{s|t} = E[(x_s - x_{s|t})(x_s - x_{s|t})^\top \mid y_1, \cdots, y_t].$$

2.1 Kalman filter

We present the Kalman filter algorithm for the Gaussian linear state space model (3) and (4).

The Kalman filter computes the one-step ahead predictive distribution $\mathrm{N}(x_{t|t-1}, \Sigma_{t|t-1})$, and the filtering distribution $\mathrm{N}(x_{t|t}, \Sigma_{t|t})$ sequentially, in the order

$$(x_{1|0}, \Sigma_{1|0}) \to (x_{1|1}, \Sigma_{1|1}) \to (x_{2|1}, \Sigma_{2|1}) \to (x_{2|2}, \Sigma_{2|2}) \to \cdots \to (x_{t|t}, \Sigma_{t|t}).$$

From the filtering distribution $\mathrm{N}(x_{t-1|t-1}, \Sigma_{t-1|t-1})$, the one-step ahead predictive distribution $\mathrm{N}(x_{t|t-1}, \Sigma_{t|t-1})$ is computed as

$$x_{t|t-1} = F x_{t-1|t-1}, \quad \Sigma_{t|t-1} = F \Sigma_{t-1|t-1} F^\top + Q.$$

From the one-step ahead predictive distribution $\mathrm{N}(x_{t|t-1}, \Sigma_{t|t-1})$ and y_t, the filtering distribution $\mathrm{N}(x_{t|t}, \Sigma_{t|t})$ is computed as

$$x_{t|t} = x_{t|t-1} + K_t(y_t - H x_{t|t-1}), \quad \Sigma_{t|t} = \Sigma_{t|t-1} - K_t H \Sigma_{t|t-1},$$

where

$$K_t = \Sigma_{t|t-1} H^\top (H \Sigma_{t|t-1} H^\top + R)^{-1}$$

is called the Kalman gain.

2.2 Kalman smoother

We present the fixed-interval Kalman smoother algorithm for the Gaussian linear state space model (3) and (4).

The fixed-interval Kalman smoother computes the smoothing distribution $\mathrm{N}(x_{s|N}, \Sigma_{s|N})$ sequentially in the order

$$(x_{t|t}, \Sigma_{t|t}) \to (x_{N-1|N}, \Sigma_{N-1|N}) \to \cdots \to (x_{1|N}, \Sigma_{1|N}).$$

It requires the one-step ahead predictive distribution $\mathrm{N}(x_{t|t-1}, \Sigma_{t|t-1})$ and the filtering distribution $\mathrm{N}(x_{t|t}, \Sigma_{t|t})$, which are obtained using the Kalman filter.

From the smoothing distribution $\mathrm{N}(x_{t+1|N}, \Sigma_{t+1|N})$, the smoothing distribution $\mathrm{N}(x_{t|N}, \Sigma_{t|N})$ is computed as

$$x_{t|N} = x_{t|t} + A_t(x_{t+1|N} - x_{t+1|t}), \quad \Sigma_{t|N} = \Sigma_{t|t} - A_t(\Sigma_{t+1|N} - \Sigma_{t+1|t})A_t^\top,$$

where

$$A_t = \Sigma_{t|t} F^\top \Sigma_{t+1|t}^{-1}.$$

We note that $\Sigma_{t|N}$ does not depend on y. In particular, it is time-reversible: $\Sigma_{t|N} = \Sigma_{N-t+1|N}$.

3 Hessian computation and confidence interval

Here, we present an extension of the Kalman filter algorithm for computing the gradient and Hessian of the log-likelihood of the Gaussian linear state space models. It is a generalization of the algorithm in [5] to multi-dimensional observations. We also explain the method for constructing confidence intervals by using the computed Hessian.

Consider a Gaussian linear state space model

$$\begin{aligned} x_{t+1} &= F(\theta)x_t + v_t, \quad v_t \sim \mathrm{N}_n(0, Q(\theta)), \\ y_t &= H(\theta)x_t + w_t, \quad w_t \sim \mathrm{N}_m(0, R(\theta)), \end{aligned}$$

where $x_t \in \mathbb{R}^n$, $y_t \in \mathbb{R}^m$, $F \in \mathbb{R}^{n\times n}$, and $H \in \mathbb{R}^{m\times n}$. In the following, we omit the argument θ for simplicity. The one-step ahead predictive distribution of y_t is

$$p(y_t \mid y_1, \dots, y_{t-1}, \theta) = \frac{1}{(2\pi)^{m/2}(\det R_t)^{1/2}} \exp\left(-\frac{1}{2}\varepsilon_t^\top R_t^{-1} \varepsilon_t\right),$$

where ε_t and R_t are the one-step ahead prediction error and its covariance defined by

$$\varepsilon_t = y_t - Hx_{t|t-1}, \quad R_t = H\Sigma_{t|t-1}H^\top + R. \tag{5}$$

Therefore, the log-likelihood is given by

$$\begin{aligned} \log L(\theta) &= \sum_{t=1}^N \log p(y_t \mid y_1, \dots, y_{t-1}, \theta) \\ &= -\frac{N}{2}\log(2\pi) - \frac{1}{2}\sum_{t=1}^N (\log\det R_t + \varepsilon_t^\top R_t^{-1} \varepsilon_t). \end{aligned} \tag{6}$$

3.1 Gradient computation

By differentiating (6), the gradient of the log-likelihood is

$$\frac{\partial}{\partial\theta_i}\log L(\theta) = -\frac{1}{2}\sum_{t=1}^{N}\sum_{t=1}^{N}\left(\mathrm{tr}\left(R_t^{-1}\frac{\partial R_t}{\partial\theta_i}\right) + 2\varepsilon_t^\top R_t^{-1}\frac{\partial\varepsilon_t}{\partial\theta_i} - \varepsilon_t^\top R_t^{-1}\frac{\partial R_t}{\partial\theta_i}R_t^{-1}\varepsilon_t\right), \tag{7}$$

where, from (5),

$$\frac{\partial\varepsilon_t}{\partial\theta_i} = -\frac{\partial H}{\partial\theta_i}x_{t|t-1} - H\frac{\partial x_{t|t-1}}{\partial\theta_i}, \tag{8}$$

$$\frac{\partial R_t}{\partial\theta_i} = \frac{\partial H}{\partial\theta_i}\Sigma_{t|t-1}H^\top + H\frac{\partial\Sigma_{t|t-1}}{\partial\theta_i}H^\top + H\Sigma_{t|t-1}\frac{\partial H^\top}{\partial\theta_i} + \frac{\partial R}{\partial\theta_i}. \tag{9}$$

From the Kalman filter formula, the required derivatives are computed sequentially as

$$\frac{\partial x_{t|t-1}}{\partial\theta_i} = \frac{\partial F}{\partial\theta_i}x_{t-1|t-1} + F\frac{\partial x_{t-1|t-1}}{\partial\theta_i}, \tag{10}$$

$$\frac{\partial\Sigma_{t|t-1}}{\partial\theta_i} = \frac{\partial F}{\partial\theta_i}\Sigma_{t-1|t-1}F^\top + F\frac{\partial\Sigma_{t-1|t-1}}{\partial\theta_i}F^\top + F\Sigma_{t-1|t-1}\frac{\partial F^\top}{\partial\theta_i} + \frac{\partial Q}{\partial\theta_i}, \tag{11}$$

and

$$\frac{\partial K_t}{\partial\theta_i} = \frac{\partial\Sigma_{t|t-1}}{\partial\theta_i}H^\top R_t^{-1} + \Sigma_{t|t-1}\frac{\partial H^\top}{\partial\theta_i}R_t^{-1} - \Sigma_{t|t-1}H^\top R_t^{-1}\frac{\partial R_t}{\partial\theta_i}R_t^{-1}, \tag{12}$$

$$\frac{\partial x_{t|t}}{\partial\theta_i} = \frac{\partial x_{t|t-1}}{\partial\theta_i} + \frac{\partial K_t}{\partial\theta_i}\varepsilon_t + K_t\frac{\partial\varepsilon_t}{\partial\theta_i}, \tag{13}$$

$$\frac{\partial\Sigma_{t|t}}{\partial\theta_i} = \frac{\partial\Sigma_{t|t-1}}{\partial\theta_i} - \frac{\partial K_t}{\partial\theta_i}H\Sigma_{t|t-1} - K_t\frac{\partial H}{\partial\theta_i}\Sigma_{t|t-1} - K_tH\frac{\partial\Sigma_{t|t-1}}{\partial\theta_i}, \tag{14}$$

where $K_t = \Sigma_{t|t-1}H^\top R_t^{-1}$ is the Kalman gain.

3.2 Hessian computation

By differentiating (7) and using

$$\frac{\partial}{\partial\theta_i}(R_t^{-1}) = -R_t^{-1}\frac{\partial R_t}{\partial\theta_i}R_t^{-1},$$

the Hessian of the log-likelihood is

$$\begin{aligned}\frac{\partial^2}{\partial\theta_i\partial\theta_j}\log L(\theta) = -\frac{1}{2}\sum_{t=1}^{N}\Bigg(&\mathrm{tr}\left(-R_t^{-1}\frac{\partial R_t}{\partial\theta_j}R_t^{-1}\frac{\partial^2 R_t}{\partial\theta_i} + R_t^{-1}\frac{\partial^2 R_t}{\partial\theta_i\partial\theta_j}\right)\\
&+ 2\frac{\partial\varepsilon_t^\top}{\partial\theta_j}R_t^{-1}\frac{\partial\varepsilon_t}{\partial\theta_i} - 2\varepsilon_t^\top R_t^{-1}\frac{\partial R_t}{\partial\theta_j}R_t^{-1}\frac{\partial\varepsilon_t}{\partial\theta_i} + 2\varepsilon_t^\top R_t^{-1}\frac{\partial^2\varepsilon_t}{\partial\theta_i\partial\theta_j}\\
&- 2\frac{\partial\varepsilon_t^\top}{\partial\theta_j}R_t^{-1}\frac{\partial R_t}{\partial\theta_i}R_t^{-1}\varepsilon_t + 2\varepsilon_t^\top R_t^{-1}\frac{\partial R_t}{\partial\theta_j}R_t^{-1}\frac{\partial R_t}{\partial\theta_i}R_t^{-1}\varepsilon_t\\
&-\varepsilon_t^\top R_t^{-1}\frac{\partial^2 R_t}{\partial\theta_i\partial\theta_j}R_t^{-1}\varepsilon_t\Bigg),\end{aligned}$$

where, from (9),

$$
\begin{aligned}
\frac{\partial^2 \varepsilon_t}{\partial\theta_i\partial\theta_j} =& -\frac{\partial^2 H}{\partial\theta_i\partial\theta_j} x_{t|t-1} - \frac{\partial H}{\partial\theta_i}\frac{\partial x_{t|t-1}}{\partial\theta_j} - \frac{\partial H}{\partial\theta_j}\frac{\partial x_{t|t-1}}{\partial\theta_i} - H\frac{\partial^2 x_{t|t-1}}{\partial\theta_i\partial\theta_j},\\
\frac{\partial^2 R_t}{\partial\theta_i\partial\theta_j} =& \frac{\partial^2 H}{\partial\theta_i\partial\theta_j}\Sigma_{t|t-1}H^\top + \frac{\partial H}{\partial\theta_i}\frac{\partial \Sigma_{t|t-1}}{\partial\theta_j}H^\top + \frac{\partial H}{\partial\theta_i}\Sigma_{t|t-1}\frac{\partial H^\top}{\partial\theta_j}\\
&+ \frac{\partial H}{\partial\theta_j}\frac{\partial \Sigma_{t|t-1}}{\partial\theta_i}H^\top + H\frac{\partial^2 \Sigma_{t|t-1}}{\partial\theta_i\partial\theta_j}H^\top + H\frac{\partial \Sigma_{t|t-1}}{\partial\theta_i}\frac{\partial H^\top}{\partial\theta_j}\\
&+ \frac{\partial H}{\partial\theta_j}\Sigma_{t|t-1}\frac{\partial H^\top}{\partial\theta_i} + H\frac{\partial \Sigma_{t|t-1}}{\partial\theta_j}\frac{\partial H^\top}{\partial\theta_i} + H\Sigma_{t|t-1}\frac{\partial^2 H^\top}{\partial\theta_i\partial\theta_j}\\
&+ \frac{\partial^2 R}{\partial\theta_i\partial\theta_j}.
\end{aligned}
$$

From (10)-(14), the required derivatives are computed sequentially as

$$
\begin{aligned}
\frac{\partial^2 x_{t|t-1}}{\partial\theta_i\partial\theta_j} =& \frac{\partial^2 F}{\partial\theta_i\partial\theta_j}x_{t-1|t-1} + \frac{\partial F}{\partial\theta_i}\frac{\partial x_{t-1|t-1}}{\partial\theta_j}\\
&+ \frac{\partial F}{\partial\theta_j}\frac{\partial x_{t-1|t-1}}{\partial\theta_i} + F\frac{\partial^2 x_{t-1|t-1}}{\partial\theta_i\partial\theta_j},\\
\frac{\partial^2 \Sigma_{t|t-1}}{\partial\theta_i\partial\theta_j} =& \frac{\partial^2 F}{\partial\theta_i\partial\theta_j}\Sigma_{t-1|t-1}F^\top + \frac{\partial F}{\partial\theta_i}\frac{\partial \Sigma_{t-1|t-1}}{\partial\theta_j}F^\top + \frac{\partial F}{\partial\theta_i}\Sigma_{t-1|t-1}\frac{\partial F^\top}{\partial\theta_j}\\
&+ \frac{\partial F}{\partial\theta_j}\frac{\partial \Sigma_{t-1|t-1}}{\partial\theta_i}F^\top + F\frac{\partial^2 \Sigma_{t-1|t-1}}{\partial\theta_i\partial\theta_j}F^\top + F\frac{\partial \Sigma_{t-1|t-1}}{\partial\theta_i}\frac{\partial F^\top}{\partial\theta_j}\\
&+ \frac{\partial F}{\partial\theta_j}\Sigma_{t-1|t-1}\frac{\partial F^\top}{\partial\theta_i} + F\frac{\partial \Sigma_{t-1|t-1}}{\partial\theta_j}\frac{\partial F^\top}{\partial\theta_i} + F\Sigma_{t-1|t-1}\frac{\partial^2 F^\top}{\partial\theta_i\partial\theta_j}\\
&+ \frac{\partial^2 Q}{\partial\theta_i\partial\theta_j},
\end{aligned}
$$

and

$$
\begin{aligned}
\frac{\partial^2 K_t}{\partial\theta_i\partial\theta_j} =& \frac{\partial^2\Sigma_{t|t-1}}{\partial\theta_i\partial\theta_j}H^\top R_t^{-1} + \frac{\partial\Sigma_{t|t-1}}{\partial\theta_i}\frac{\partial H^\top}{\partial\theta_j}R_t^{-1} - \frac{\partial\Sigma_{t|t-1}}{\partial\theta_i}H^\top R_t^{-1}\frac{\partial R_t}{\partial\theta_j}R_t^{-1} \\
&+ \frac{\partial\Sigma_{t|t-1}}{\partial\theta_j}\frac{\partial H^\top}{\partial\theta_i}R_t^{-1} + \Sigma_{t|t-1}\frac{\partial^2 H^\top}{\partial\theta_i\partial\theta_j}R_t^{-1} - \Sigma_{t|t-1}\frac{\partial H^\top}{\partial\theta_i}R_t^{-1}\frac{\partial R_t}{\partial\theta_j}R_t^{-1} \\
&- \frac{\partial\Sigma_{t|t-1}}{\partial\theta_j}H^\top R_t^{-1}\frac{\partial R_t}{\partial\theta_i}R_t^{-1} - \Sigma_{t|t-1}\frac{\partial H^\top}{\partial\theta_j}R_t^{-1}\frac{\partial R_t}{\partial\theta_i}R_t^{-1} \\
&+ \Sigma_{t|t-1}H^\top R_t^{-1}\frac{\partial R_t}{\partial\theta_j}R_t^{-1}\frac{\partial R_t}{\partial\theta_i}R_t^{-1} - \Sigma_{t|t-1}H^\top R_t^{-1}\frac{\partial^2 R_t}{\partial\theta_i\partial\theta_j}R_t^{-1} \\
&+ \Sigma_{t|t-1}H^\top R_t^{-1}\frac{\partial R_t}{\partial\theta_i}R_t^{-1}\frac{\partial R_t}{\partial\theta_j}R_t^{-1}, \\
\frac{\partial^2 x_{t|t}}{\partial\theta_i\partial\theta_j} =& \frac{\partial^2 x_{t|t-1}}{\partial\theta_i\partial\theta_j} + \frac{\partial^2 K_t}{\partial\theta_i\partial\theta_j}\varepsilon_t + \frac{\partial K_t}{\partial\theta_i}\frac{\partial\varepsilon_t}{\partial\theta_j} + \frac{\partial K_t}{\partial\theta_j}\frac{\partial\varepsilon_t}{\partial\theta_i} + K_t\frac{\partial^2\varepsilon_t}{\partial\theta_i\partial\theta_j} \\
\frac{\partial^2\Sigma_{t|t}}{\partial\theta_i\partial\theta_j} =& \frac{\partial^2\Sigma_{t|t-1}}{\partial\theta_i\partial\theta_j} - \frac{\partial^2 K_t}{\partial\theta_i\partial\theta_j}H\Sigma_{t|t-1} - \frac{\partial K_t}{\partial\theta_i}\frac{\partial H}{\partial\theta_j}\Sigma_{t|t-1} - \frac{\partial K_t}{\partial\theta_i}H\frac{\partial\Sigma_{t|t-1}}{\partial\theta_j} \\
&- \frac{\partial K_t}{\partial\theta_j}\frac{\partial H}{\partial\theta_i}\Sigma_{t|t-1} - K_t\frac{\partial^2 H}{\partial\theta_i\partial\theta_j}\Sigma_{t|t-1} - K_t\frac{\partial H}{\partial\theta_i}\frac{\partial\Sigma_{t|t-1}}{\partial\theta_j} \\
&- \frac{\partial K_t}{\partial\theta_j}H\frac{\partial\Sigma_{t|t-1}}{\partial\theta_i} - K_t\frac{\partial H}{\partial\theta_j}\frac{\partial\Sigma_{t|t-1}}{\partial\theta_i} - K_t H\frac{\partial^2\Sigma_{t|t-1}}{\partial\theta\partial\theta_i^\top}.
\end{aligned}
$$

3.3 Application to OSC-DECOMP

The state space model of OSC-DECOMP corresponds to

$$
F = \begin{pmatrix} F_1 & O & \cdots & O \\ O & F_2 & \cdots & O \\ \vdots & \vdots & \ddots & \vdots \\ O & O & \cdots & F_K \end{pmatrix}, \quad F_k = a_k \begin{pmatrix} \cos(2\pi f_k\Delta t) & -\sin(2\pi f_k\Delta t) \\ \sin(2\pi f_k\Delta t) & \cos(2\pi f_k\Delta t) \end{pmatrix},
$$

$$
Q = \begin{pmatrix} Q_1 & O & \cdots & O \\ O & Q_2 & \cdots & O \\ \vdots & \vdots & \ddots & \vdots \\ O & O & \cdots & Q_K \end{pmatrix}, \quad Q_k = \begin{pmatrix} \sigma_k^2 & 0 \\ 0 & \sigma_k^2 \end{pmatrix},
$$

$$
H = \begin{pmatrix} 1 & 0 & 1 & 0 & \cdots & 1 & 0 \\ c_{21,1} & c_{21,2} & c_{22,1} & c_{22,2} & \cdots & c_{2K,1} & c_{2K,2} \\ \vdots & \vdots & \vdots & \vdots & \ddots & \vdots & \\ c_{J1,1} & c_{J1,2} & c_{J2,1} & c_{J2,2} & \cdots & c_{JK,1} & c_{JK,2} \end{pmatrix}, \quad R = \tau^2 I_J.
$$

The parameter is $\theta = (a_1, \dots, a_K, f_1, \dots, f_K, \sigma_1^2, \dots, \sigma_K^2, c_{21,1}, \dots, c_{JK,2}, \tau^2)$. Thus, the nonzero derivatives of F, Q, H, R are

$$\frac{\partial F_k}{\partial a_k} = \begin{pmatrix} \cos(2\pi f_k \Delta t) & -\sin(2\pi f_k \Delta t) \\ \sin(2\pi f_k \Delta t) & \cos(2\pi f_k \Delta t) \end{pmatrix},$$
$$\frac{\partial F_k}{\partial f_k} = 2\pi a_k \Delta t \begin{pmatrix} -\sin(2\pi f_k \Delta t) & -\cos(2\pi f_k \Delta t) \\ \cos(2\pi f_k \Delta t) & -\sin(2\pi f_k \Delta t) \end{pmatrix},$$
$$\frac{\partial^2 F_k}{\partial a_k \partial f_k} = 2\pi \Delta t \begin{pmatrix} -\sin(2\pi f_k \Delta t) & -\cos(2\pi f_k \Delta t) \\ \cos(2\pi f_k \Delta t) & -\sin(2\pi f_k \Delta t) \end{pmatrix},$$
$$\frac{\partial^2 F_k}{\partial f_k^2} = 4\pi^2 a_k (\Delta t)^2 \begin{pmatrix} -\cos(2\pi f_k \Delta t) & \sin(2\pi f_k \Delta t) \\ -\sin(2\pi f_k \Delta t) & -\cos(2\pi f_k \Delta t) \end{pmatrix},$$
$$\frac{\partial Q_k}{\partial (\sigma_k^2)} = \begin{pmatrix} 1 & 0 \\ 0 & 1 \end{pmatrix}, \quad \frac{\partial H_{j,2k+l-2}}{\partial c_{jk,l}} = 1, \quad \frac{\partial R}{\partial (\tau^2)} = I_J.$$

Also, from the stationary distribution

$$x_{1|0} = 0, \quad (V_{1|0})_{2k-1,2k-1} = (V_{1|0})_{2k,2k} = \frac{\sigma_k^2}{1 - a_k^2}, \quad (V_{1|0})_{ij} = 0 \ (i \neq j),$$

the initial gradient and Hessian are

$$\frac{\partial x_{1|0}}{\partial \theta_i} = 0, \quad \frac{\partial^2 x_{1|0}}{\partial \theta_i \partial \theta_j} = 0,$$
$$\frac{\partial (V_{1|0})_{2k-1,2k-1}}{\partial a_k} = \frac{\partial (V_{1|0})_{2k,2k}}{\partial a_k} = \frac{2 a_k \sigma_k^2}{(1 - a_k^2)^2},$$
$$\frac{\partial (V_{1|0})_{2k-1,2k-1}}{\partial (\sigma_k^2)} = \frac{\partial (V_{1|0})_{2k,2k}}{\partial (\sigma_k^2)} = \frac{1}{1 - a_k^2},$$
$$\frac{\partial^2 (V_{1|0})_{2k-1,2k-1}}{\partial a_k^2} = \frac{\partial^2 (V_{1|0})_{2k,2k}}{\partial a_k^2} = \frac{2(3a_k^2 + 1)\sigma_k^2}{(1 - a_k^2)^3},$$
$$\frac{\partial^2 (V_{1|0})_{2k-1,2k-1}}{\partial a_k \partial (\sigma_k^2)} = \frac{\partial^2 (V_{1|0})_{2k,2k}}{\partial a_k \partial (\sigma_k^2)} = \frac{2 a_k}{(1 - a_k^2)^2},$$

where other derivatives of $V_{1|0}$ are zero.

3.4 Confidence intervals

Let

$$H(\theta) = -\left(\frac{\partial^2}{\partial \theta_i \partial \theta_j} \log L(\tilde{\theta}) \right)_{ij}$$

be the Hessian of the negative log-likelihood. For the maximum likelihood estimate $\hat{\theta}$, the matrix $H(\hat{\theta})$ is often called the observed Fisher information [6]. From the theory of asymptotic statistics [7], the asymptotic covariance matrix of $\hat{\theta}$ can be approximated by $V = H(\hat{\theta})^{-1}$. Therefore, the 95 % confidence interval of θ_i can be constructed as

$$\left[\hat{\theta}_i - 1.96\sqrt{V_{ii}}, \hat{\theta}_i + 1.96\sqrt{V_{ii}} \right].$$

For the phase difference $\phi_{jk} = \arg(c_{jk,1}+c_{jk,2}\sqrt{-1})$, its confidence interval can be computed by using the delta method [7]. Let $V_{(jk)}$ be the 2×2 submarix of V corresponding to $c_{jk,1}$ and $c_{jk,2}$. Then, from

$$\frac{\partial \phi_{jk}}{\partial c_{jk,1}} = -\frac{c_{jk,2}}{c_{jk,1}^2 + c_{jk,2}^2}, \quad \frac{\partial \phi_{jk}}{\partial c_{jk,2}} = \frac{c_{jk,1}}{c_{jk,1}^2 + c_{jk,2}^2},$$

the asymptotic variance of $\hat{\phi}_{jk} = \arg(\hat{c}_{jk,1} + \hat{c}_{jk,2}\sqrt{-1})$ is approximated by

$$v_{(jk)} = \frac{\hat{c}_{jk,2}^2}{(\hat{c}_{jk,1}^2 + \hat{c}_{jk,2}^2)^2}(V_{(jk)})_{11} - 2\frac{\hat{c}_{jk,1}\hat{c}_{jk,2}}{(\hat{c}_{jk,1}^2 + \hat{c}_{jk,2}^2)^2}(V_{(jk)})_{12} + \frac{\hat{c}_{jk,1}^2}{(\hat{c}_{jk,1}^2 + \hat{c}_{jk,2}^2)^2}(V_{(jk)})_{22}.$$

Therefore, the 95 % confidence interval of ϕ_{jk} can be constructed as

$$\left[\hat{\phi}_{jk} - 1.96\sqrt{v_{(jk)}}, \hat{\phi}_{jk} + 1.96\sqrt{v_{(jk)}}\right].$$

References

[1] Durbin, J. & Koopman, S. J. (2012) *Time Series Analysis by State Space Methods.* Oxford: Oxford University Press.

[2] Kitagawa, G. (2010). *Introduction to Time Series Modeling.* London: Chapman & Hall.

[3] Kitagawa, G. & Gersch, W. (1984). A smoothness priors-state space modeling of time series with trend and seasonality. *Journal of the American Statistical Association* **79**, 378–389.

[4] West, M. (1997). Time series decomposition. *Biometrika* **84**, 489–494.

[5] Kitagawa, G. (2020). Computation of the gradient and Hessian of the log-likelihood of the state-space model by the Kalman filter. arXiv:2011.09638.

[6] Efron, B. & Hinkley, D. V. (1978). Assessing the accuracy of the maximum likelihood estimator: Observed versus expected Fisher information. *Biometrika* **65**, 457–483.

[7] van der Vaart, A. W. (1998). *Asymptotic Statistics.* Cambridge University Press.
